# Systems pathology by multiplexed immunohistochemistry and whole-slide digital image analysis

**DOI:** 10.1038/s41598-017-15798-4

**Published:** 2017-11-14

**Authors:** Sami Blom, Lassi Paavolainen, Dmitrii Bychkov, Riku Turkki, Petra Mäki-Teeri, Annabrita Hemmes, Katja Välimäki, Johan Lundin, Olli Kallioniemi, Teijo Pellinen

**Affiliations:** 1 Institute for Molecular Medicine Finland FIMM, University of Helsinki, Helsinki, Finland; 2 Science for Life Laboratory, Karolinska Institutet, Department of Oncology and Pathology, Solna, Sweden

## Abstract

The paradigm of molecular histopathology is shifting from a single-marker immunohistochemistry towards multiplexed detection of markers to better understand the complex pathological processes. However, there are no systems allowing multiplexed IHC (mIHC) with high-resolution whole-slide tissue imaging and analysis, yet providing feasible throughput for routine use. We present an mIHC platform combining fluorescent and chromogenic staining with automated whole-slide imaging and integrated whole-slide image analysis, enabling simultaneous detection of six protein markers and nuclei, and automatic quantification and classification of hundreds of thousands of cells *in situ* in formalin-fixed paraffin-embedded tissues. In the first proof-of-concept, we detected immune cells at cell-level resolution (n = 128,894 cells) in human prostate cancer, and analysed T cell subpopulations in different tumour compartments (epithelium vs. stroma). In the second proof-of-concept, we demonstrated an automatic classification of epithelial cell populations (n = 83,558) and glands (benign vs. cancer) in prostate cancer with simultaneous analysis of androgen receptor (AR) and alpha-methylacyl-CoA (AMACR) expression at cell-level resolution. We conclude that the open-source combination of 8-plex mIHC detection, whole-slide image acquisition and analysis provides a robust tool allowing quantitative, spatially resolved whole-slide tissue cytometry directly in formalin-fixed human tumour tissues for improved characterization of histology and the tumour microenvironment.

## Introduction

It is important to understand the spatial cellular composition and heterogeneity of tissues, especially in cancer where cell subpopulations and the tumour microenvironment provide insights about the biology and clinical progression of the disease. The standard method for detecting proteins *in situ* is immunohistochemistry (IHC) on thin sections of formalin-fixed paraffin-embedded (FFPE) tissue followed by a visual assessment of antibody reactivity. However, as the analysis of multiple markers is performed on consecutive sections, it is impossible to assess co-localization of markers at single cell level, which radically limits accurate classification of cells that require detection of multiple markers (e.g. different subtypes of immune cells). In order to better understand the pathological processes and deliver more accurate prognostics and patient stratification for treatments, tumours should be characterized more comprehensively, integrating cell-level information with context specific information of the microenvironment. However, the limitations of the traditional IHC have impeded the evolution of histopathology towards truly multi-parametric analysis of whole tissue sections.

In contrast to conventional IHC, multiplexed IHC (mIHC) enables multi-parametric readouts from a single tissue section. The current state-of-the-art employ either fluorescence^1–9^ or mass spectrometry^10–12^ detection. Although various sophisticated mIHC methods are available for FFPE material, the current applications have limited scalability and throughput, because, although showing high level of multiplexing, the analysis is limited to small region-of-interests and/or limited number of fields-of-views^2,4,6–9,13–15^. For example, >5-plex fluorescence assays utilizing multispectral imaging are slow in terms of image acquisition. One solution to overcome this limitation is to utilize a “hotspot” imaging where a low-resolution scan of whole tissue is performed first followed by a subsequent “hotspot” analysis at higher resolution^1,16^. Nevertheless, this assay design does not allow true whole-slide analytics. Other promising technologies for fluorescence mIHC rely on dye cycling, namely MxIF^5^ and CycIF^17^, which utilize fluorochrome bleaching and/or antibody stripping between staining cycles. The level of multiplexing of these “temporally” resolved assays is much higher than of the spectrally resolved assays, even up to 61 markers per section^17^. However, major drawbacks of dye cycling are the laborious staining/imaging cycles^5,17^, the primary antibody labelling for direct fluorescence detection^5,17^, and potential changes of the tissue morphology and antigenicity due to the repetitive exposure of the tissue to the dye bleaching and/or antibody stripping conditions^5^. In contrast to fluorescence, mass spectrometry based methods provide highly multiplexed mIHC assays^10–12,17^ omitting most of the pitfalls of fluorescence imaging. Mass spectrometry holds a great potential for the future, but the instrumentation is still expensive, not easily accessible, and the spectrometry “image” acquisition is extremely slow, even when compared to multispectral fluorescence acquisition, being impractical for routine whole-slide analytics at cell-level resolution.

Despite of the issue in terms of scalability and throughput, multiplexed IHC (mIHC) methods allow simultaneous detection and co-localization analysis of multiple markers *in situ* in the intact spatial context of tissues^1–15,17–19^. Moreover, multiplexing allows for a simple and easily automated, marker-guided tissue segmentation (e.g. epithelium vs. stroma), and provides more information from each tissue section, which may be critically important for small samples, such as needle biopsies of tissues or small metastatic tumour samples. However, as tumours often exhibit significant cellular and spatial heterogeneity, it would be important to be able to perform high-resolution, multiplexed analysis across whole-sections of tumours^20^. Hence, there is a growing need for an integrated “workhorse” mIHC system enabling not only moderate degree of multiplexing but also imaging and image analysis for high-resolution whole-slide analytics.

Here, we describe a whole-slide 8-plex mIHC platform combining fluorescence (five-channel) and chromogenic (three-channel) mIHC allowing for a quantitative whole-slide analysis of six markers and cell nuclei at cell-level resolution. Our mIHC method is based on a fixed set of secondary reagents instead of labelled primary antibodies, thus enabling rapid implementation and virtually unlimited design of custom antibody panels for different targets of interest. We also provide protocols and methods for implementing new antibodies and targets for mIHC. As a proof-of-concept, we demonstrate automated classification of epithelial and immune cells in human prostate cancer and a simultaneous marker analysis at single cell level. To our knowledge, this is the first open-source 8-plex mIHC assay design that enables whole-slide imaging with true quantitative whole-slide analysis in FFPE samples at high resolution across the whole tissue.

## Results

**Figure 1:**
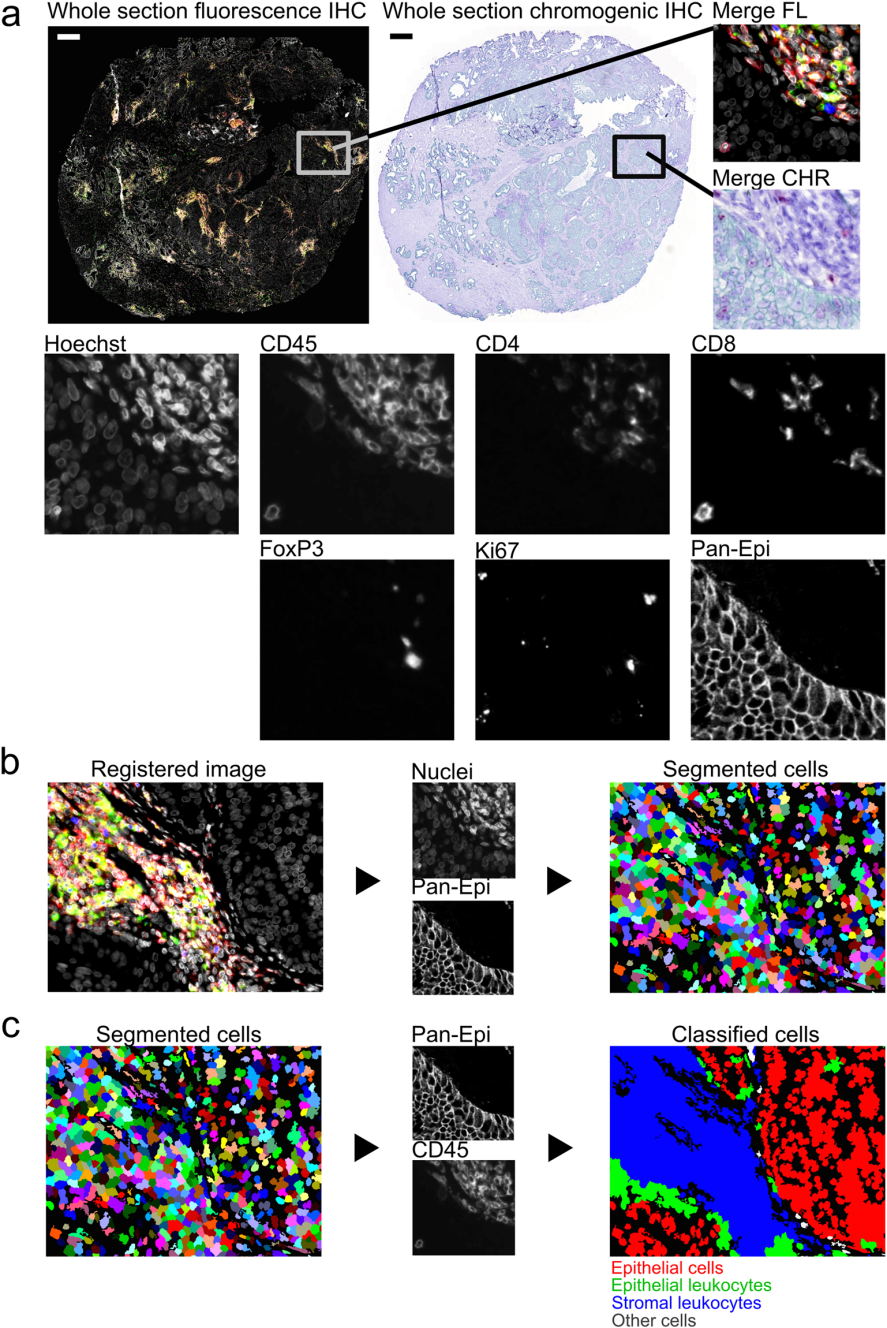
Multiplexed immunohistochemistry for immune cells in prostate tumour (patient 1). (**a**) Fluorescence (FL) and brightfield (CHR) images are acquired and (**b**) registered using nuclei information from both images (Hoechst and haematoxylin). (**c**) Cell segmentation and classification is based on nuclei, epithelium marker expression (Pan-Epi = Pan-CK + ECad), and cell-type specific marker expression (CD45 for leukocytes). Scale bar 500 µm. CHR, chromogenic; FL, fluorescence; IHC, immunohistochemistry; Pan-CK, pan-cytokeratin; Pan-Epi; pan-epithelium.

**Figure 2:**
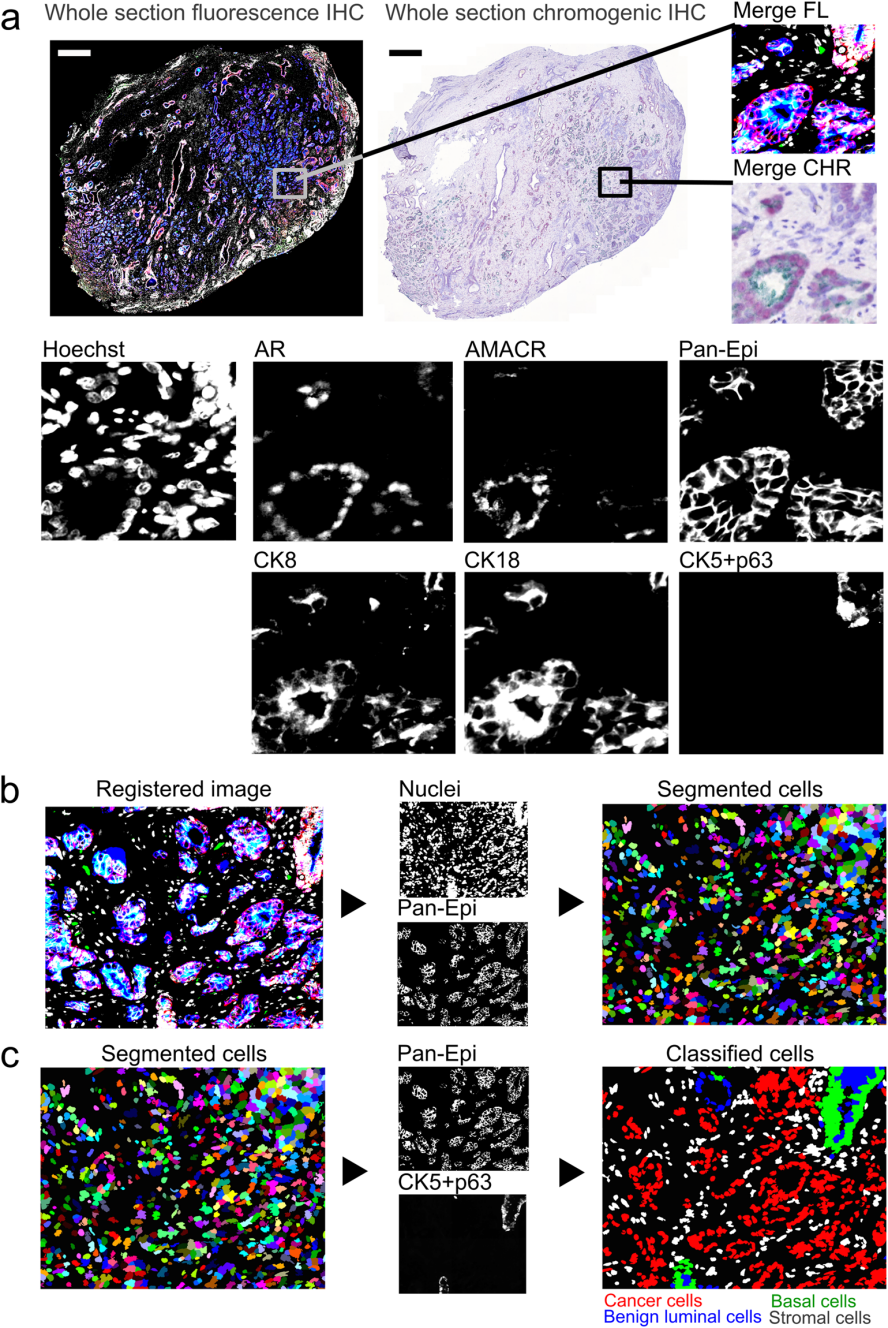
Multiplexed immunohistochemistry for epithelial cells in prostate tumour. (**a**) Fluorescence (FL) and brightfield (CHR) images were acquired and (**b**) images are registered using nuclei information from both images, namely Hoechst and haematoxylin. (**c**) Cell segmentation and classification is based on epithelium marker expression (Pan-Epi = Pan-CK + E-cadherin) and cell-type specific classifier marker expression in each prostate gland. A given gland is classified as cancerous if basal cell marker expression (CK5 + p63) is lost. Scale bar 500 µm. AMACR, alpha-methylacyl-CoA racemase; AR, androgen receptor; CK5, cytokeratin 5; CK8, cytokeratin 8; CK18, cytokeratin 18; CHR, chromogenic; FL, fluorescence; IHC, immunohistochemistry; Pan-CK, pan-cytokeratin; Pan-Epi; pan-epithelium.

As a proof-of-concept, we applied the mIHC method for the whole-slide analysis of immune cells (Fig. 1) and prostate epithelial cells (Fig. 2) in human prostate cancer (PCa) samples. The method utilizes a combination of fluorescent and chromogenic tissue staining for the simultaneous detection of six proteins and nuclei in formalin-fixed tissue section (Supplementary Fig. S1 and Supplementary Fig. S2).

We designed an antibody panel for the detection and classification of immune cells (CD45, CD4, CD8, and FoxP3), including antibodies against pan-cytokeratin (Pan-CK) and epithelial cadherin (E-cadherin) as well as Ki67 to measure cell proliferation (Immune cell panel). The other panel was designed for the characterisation of prostate epithelium and consisted of antibodies for p63, Pan-CK, CK5, CK8, CK18, alpha-methylacyl-CoA racemase (AMACR), and androgen receptor (AR) (Epithelial cell panel).

### Antibody validation for mIHC

We tested all primary antibodies used in the study in lymph node and prostate FFPE tissues. Epithelial markers CK18, CK8, CK5, p63, E-Cadherin, and Pan-CK were negative in lymph node (negative for epithelium) and positive in prostate epithelial cells. In contrast, immune cell markers CD4, CD8, CD45, and FoxP3 showed positive staining in lymph node whereas prostate epithelial cells were negative. The expression of AR was weak in some immune cells in lymph node and strong in prostate luminal epithelial cells. AMACR was expressed only in cancerous prostate cells. Ki67 was positive in lymph node germinal center but negative in well-differentiated prostate epithelium (Supplementary Fig. S3).

As the detection of the first pair of primary antibodies is based on tyramide signal amplification (TSA)^18,21,22^ followed by an inactivating heating step and additional pair of primary antibodies (see Supplementary Fig. S2), it was important to assess antibody denaturation/stripping efficacy. To test if the primary antibodies are either detached from the tissue or denatured *in situ* by heating, we incubated the tissues with bound primary antibodies in a hot buffer recapitulating the conditions in the heat-induced epitope retrieval (HIER) protocol. First, we incubated tissue-bound primary-secondary antibody complexes in hot buffer to see if the harsh conditions reduce fluorescence signal. After heating the anti-CK5/anti-rabbit-AlexaFluor555 complex (Tris-EDTA pH 9), we observed 94% of the fluorescence intensity as compared to non-heated control. After subsequent heating in glycine (pH 2), we observed 102% of the fluorescence intensity as compared to non-heated control. In case of the  anti-CK8/anti-mouse-AlexaFluor647 complex, the corresponding fluorescence was 95% and 74% (Supplementary Fig. S4). Next, we performed an assay to detect antibody denaturation, first heating the tissue sections with bound primary antibodies in hot buffer and then detecting the primary antibodies using secondary antibodies. As most primary antibodies showed complete absence of DAB signal after heat exposure even at high antibody concentrations, but fluorescence signals were not significantly diminished after heating in the earlier experiments, we conclude that the primary antibodies (and secondary antibodies) are denatured, but not detached in the HIER conditions. E-cadherin (BD Biosciences 610182) and Ki67 (Abcam 92742) antibodies, however, were not completely denatured upon the heating step (Supplementary Fig. S5). Hence, all the primary antibodies require testing for the denaturation efficacy.

### mIHC performance

Next, we tested the fluorescence mIHC in terms of imaging and signal-to-background (S/B) performance. We designed a fluorescence imaging system with labels and optical filter sets including near infrared (NIR) detection (Supplementary Table S1) to maximise spectral resolution, yielding raw image data that do not require channel unmixing after image acquisition. The pairwise signal-to-background (S/B) ratios for AlexaFluor488 (30 ms exposure in FITC channel), AlexaFluor555 (40 ms exposure in CY3 channel), AlexaFluor647 (100 ms or 50 ms exposure in CY5 channel), and AlexaFluor750 (150 ms exposure in CY7 channel) were 51, 17 (with AF488) or 25 (with AF647), 9 (with AF555) or 11 (with AF750), and 6.3, respectively. With background noise thresholding (tissue autofluorescence) the corresponding ratios were 48, 15 or 23, 7 or 10, and 3.8 (Supplementary Fig. S6).

### Immune cell classification and analysis

For the classification of immune cells in prostate cancer epithelium and stroma, we detected cells by nuclear staining, and identified leukocytes and epithelial cells using markers for pan-leukocyte (CD45) and pan-epithelium (Pan-Epi = pan-cytokeratin + E-cadherin). We classified the CD45^+^ leukocytes as stromal or epithelial depending on their location within the tissue segments, and furtheir classified each leukocyte as T regulatory (CD4^+^FoxP3^+^CD8^−^), T helper (CD4^+^FoxP3^−^CD8^−^), or T effector (CD8^+^CD4^−^FoxP3^−^) cell.

**Figure 3:**
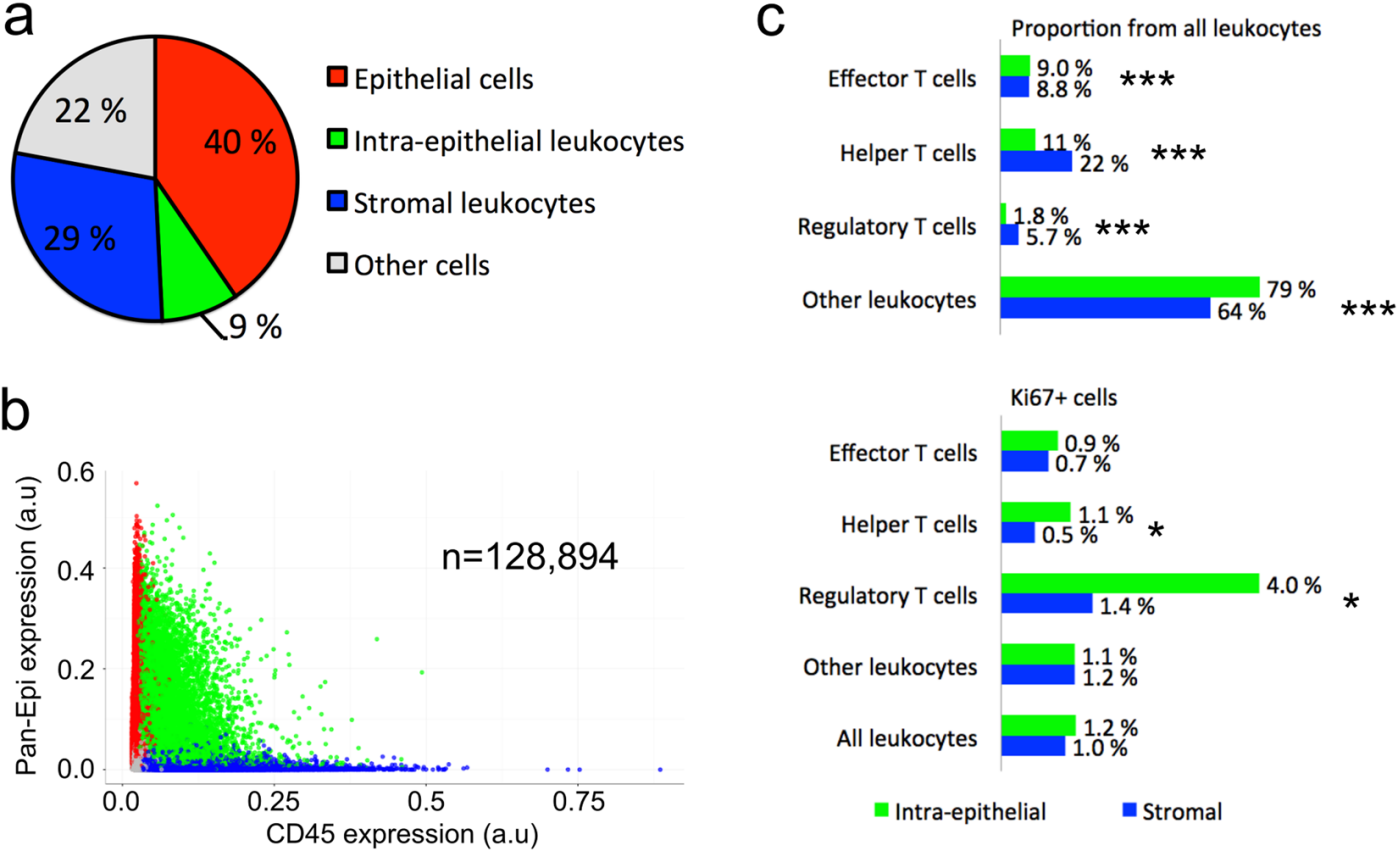
Tissue cytometry of immune cells (patient 1) in prostate cancer. (**a**) Cell class distribution and (**b**) CD45 and Pan-Epi expression in all cells (n = 128,894). (**c**) The proportion of different T cell classes from all leukocytes and proliferation (Ki67^+^) of T cells (*p < 0.05, ***p < 0.001, X^2^ exact test, two-tailed). Pan-Epi, pan-epithelium.

A total of 128,894 cells were detected in a whole section of a prostate tumour (patient 1), consisting of 38% leukocytes, 40% epithelial cells, and 22% stromal cells (Fig. 3). Among leukocytes, 24% and 76% were within the prostate epithelium or stroma, respectively. We found significantly lower proportion of regulatory T cells (Treg) (1.8% vs. 5.7%, p < 0.0005) and helper T cells (Th) (11% vs. 22%, p < 0.0005) in the epithelium as compared to the stroma, whereas the proportion of effector T cells (Te) was almost the same in the epithelium and in the stroma (9.0% vs. 8.8%, p < 0.0005). However, the proliferation rate (Ki67^+^ cells) was almost three-fold for Treg cells (4.0% vs. 1.4%, p = 0.013) and double for Th cells (1.1% vs. 0.5%, p = 0.029) in the epithelium as compared to the stroma. All marker expression values are presented in Supplementary Table S2.

### Epithelial cell classification and analysis

Next, we studied the composition of prostate tumour tissue using the Epithelial cell panel to detect prostate cells in individual prostatic glands and to automatically classify the detected cells as stromal, basal epithelial, benign luminal epithelial, or cancer cells. First, we segmented the epithelium and stroma and then classified epithelial glands as either benign or malignant based on the presence of CK5^+^p63^+^ basal cells. A gland was considered to be malignant in case of a complete absence of basal cells. Next, we segmented and classified individual cells as stromal, basal epithelial (Pan-CK^+^CK5^+^p63^+^), benign luminal epithelial (Pan-CK^+^CK5^−^p63^−^ cells located in a benign gland), or cancer cells (Pan-CK^+^CK5^−^p63^−^ cells located in a malignant gland).

**Figure 4:**
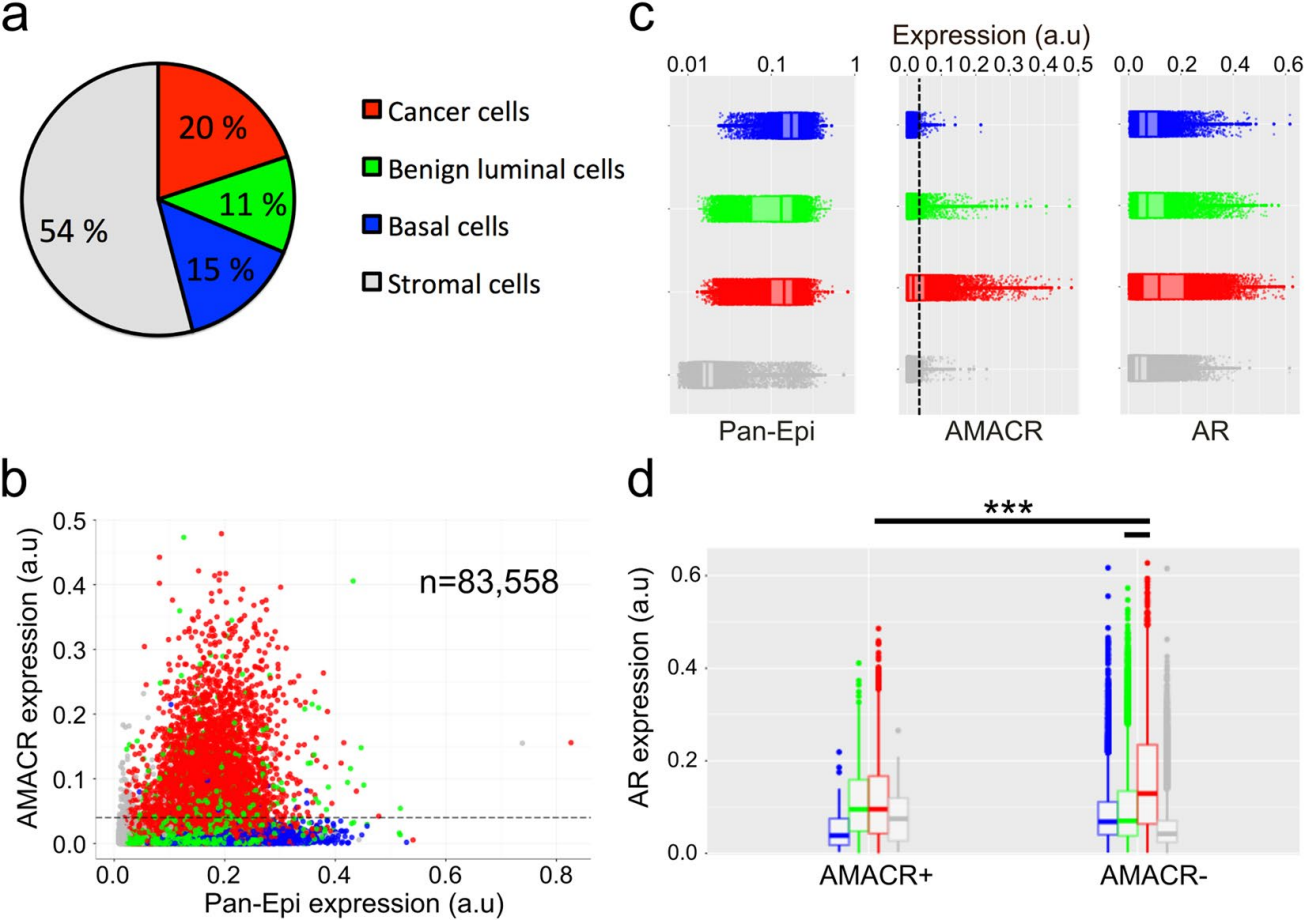
Tissue cytometry of epithelial cells (patient 2) in prostate cancer. (**a**) Cell class distribution and (**b**) AMACR and Pan-Epi expression in all cells (n = 83,558) with AMACR positivity threshold indicated (dashed line). (**c**) Expression profile of Pan-Epi, AMACR, and AR differs between the four cell classes. (**d**) AR expression in AMACR^−^ cancer cells, AMACR^+^ cancer cells, and in AMACR^−^ benign luminal epithelial cells (***p < 0.001, non-parametric Kolmogorov-Smirnov test). Boxplots indicate median and quartiles. AMACR, alpha-methylacyl-CoA racemase; AR, androgen receptor; Pan-Epi, pan-epithelium.

We detected a total of 83,558 cells in a whole-section of a prostate cancer sample (patient 2), of which 46% and 54% were epithelial and stromal cells, respectively (Fig. 4). Overall, we classified 20% of all cells as cancer, 11% as benign luminal, and 15% as basal cells. After classification, the expression of AMACR, AR, CK8, and CK18 was measured in all cells (Supplementary Table S2). The cytokeratin analysis in individual cells revealed 68% stronger expression of CK18 in prostate cancer cells (n = 16,614) as compared to benign luminal cells (n = 9,576) (0.200 vs. 0.119, p < 0.001), whereas the expression of CK8 was only 14% higher in cancer cells (0.081 vs. 0.071, p < 0.001). Interestingly, we found that AR expression was 43% higher in AMACR^−^ cancer cells (n = 11,511) than in AMACR^+^ cancer cells (n = 5,103) (0.160 vs. 0.112, p < 0.001) and 58% higher than in AMACR^−^ benign luminal cells (n = 9,068) (0.160 vs. 0.101, p < 0.0005). However, AMACR^+^ benign luminal cells (n = 508) showed similar AR expression as AMACR^+^ cancer cells (0.113 vs. 0.112, p = 0.191). Overall, we found an inverse correlation between AR and AMACR in cancer cells (Pearson r = −0.26, p < 0.00001), but not in benign luminal cells (r = −0.05, p < 0.00001). As expected, the mean expressions of the epithelial markers, CK18, CK8, and Pan-Epi were weak in stromal cells.

## Discussion

Advances in the understanding of tumour biology are rapidly changing the pathology field enabling better prognostics and new therapies. However, the tools to study cellular and molecular components of the tumour microenvironment need further development. In particular, there is a need for open-source systems and methods that would allow high-resolution whole-slide multiplexed readouts. For instance, it has been shown that analysis of small regions of interest causes significant variation and error in the assessment of molecular markers in breast cancer^20^. Although there has been a surge of novel technologies for sophisticated mIHC, the methods have limitations regarding their applicability for large-scale studies with quantitative whole-slide analysis either due to expensive instrumentation, the time needed to acquire whole-slide data, or due to antibody modifications required prior to the assay. Currently, there are no publications describing >5-plex mIHC assay design with high-resolution whole-slide imaging, open-source whole-slide image analysis at cell-level resolution, and the possibility to implement new targets rapidly (see Supplementary Table S3 for a summary).

**Figure 5:**
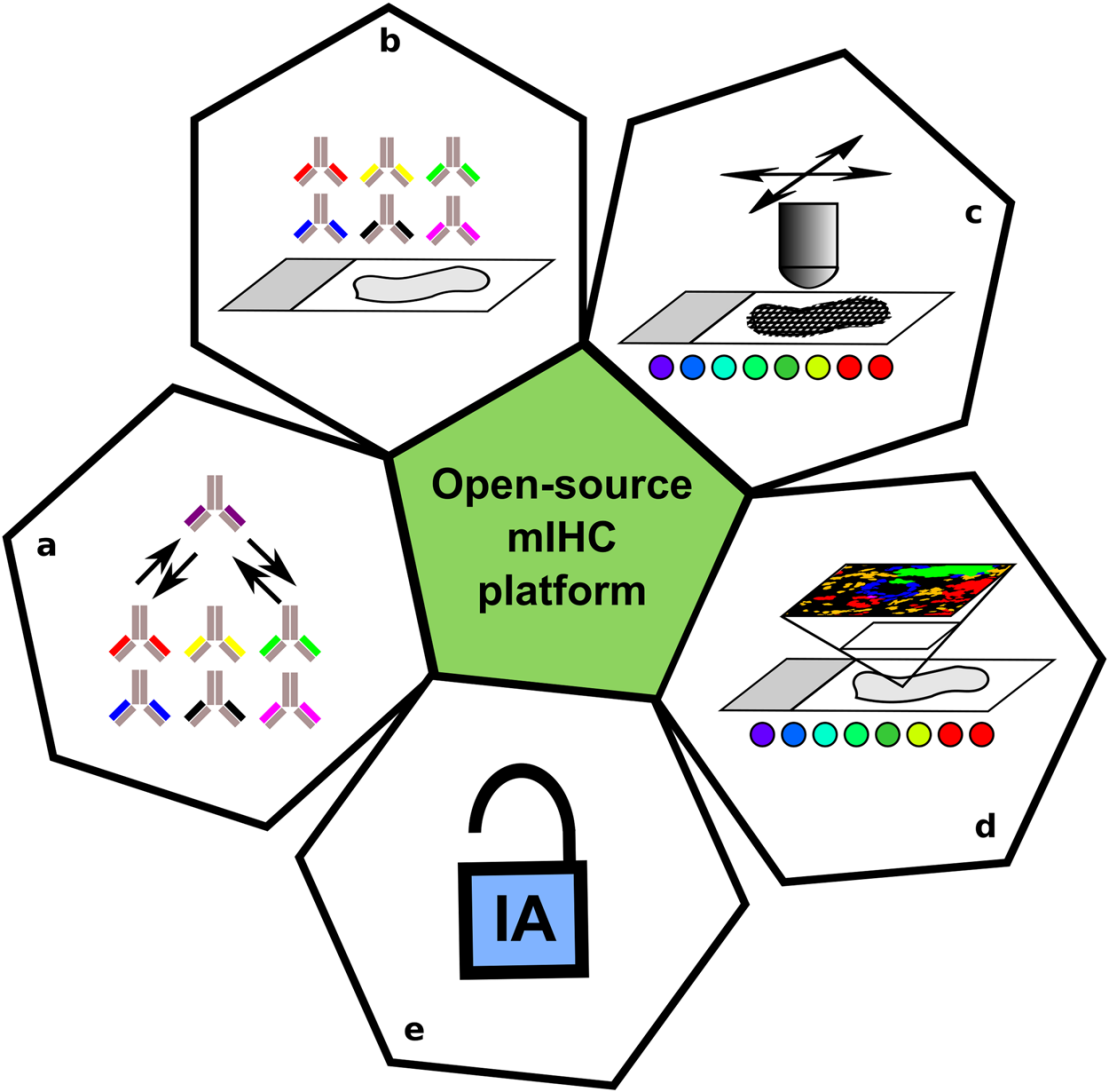
Summary of key aspects of the described mIHC platform combining (**a**) rapid implementation of new antibodies for targets of interest, (**b**) >5-plex mIHC assay design, (**c**) high-resolution whole-slide image acquisition, and (**d**,**e**) open-source high-resolution whole-slide image analysis.

Here, we present an mIHC method for the whole-slide analysis of immune cells and epithelial cells in human prostate cancer combining fluorescent and chromogenic detection of six proteins and nuclei in the same tissue section. As the system is designed for a fixed set of secondary reagents and unlabelled primary antibodies, introducing new primary antibodies is rapid allowing flexible design of custom mIHC target panels. The presented mIHC system enables not only a multiplexed analysis but also an open-source high-resolution digital image analysis across entire tissue sections (Fig. 5).

In the first proof-of-concept for immune cell detection, we detected 128,894 cells in a single whole tissue section of a prostate tumour. We found significantly less regulatory T cells and helper T cells in the tumour epithelium as compared to the stroma. Nevertheless, both cell types showed substantially higher proliferation rate in the epithelium than in the stroma, which could be a reflection of immunosuppressive microenvironment in this patient case, since regulatory T cells have been shown to suppress immune system in cancer^23^. However, as we studied only a single patient case to present the technical proof-of-concept, no biological conclusions can be drawn. Nevertheless, this type of single immune cell classification and analysis in the different tissue compartments is only possible by using multiplexed detection in the intact tissue context on whole-slides.

In the second proof-of-concept, we report an automatic classification of individual prostatic epithelial cells as benign or cancerous based on the epithelial marker profiling. Here, the detection of basal cells using CK5 and p63 is important, as the loss of basal cells is a diagnostic hallmark of PCa^24^. In addition, several prognostic biomarkers have been suggested for PCa^25,26^, but few have been translated in the clinic. Thus, PCa diagnostics and prognostics could benefit from an automatic whole-slide analysis combining readouts for the absence of basal cells and for the expression of key biomarkers, such as AMACR and AR, at single cell level. To demonstrate this, we detected and automatically classified 83,558 cells in the whole tissue section as stromal, basal epithelial, benign luminal epithelial, or cancer cells, and then measured the expression of CK8, CK18, AMACR and AR in all the cells quantitatively. We found that the expression of AR and CK18 were elevated in cancer cells as compared to benign luminal cells or basal cells, which has been also reported earlier^27,28^. However, the expression of AR in AMACR^−^ cancer cells was remarkably stronger than in either AMACR^+^ cancer cells or AMACR^−^ benign epithelial cells, whereas AMACR^+^ cancer cells and AMACR^+^ benign luminal cells showed similar AR expression. As AMACR positivity in the prostate epithelial cells is a proposed marker for a pre-malignant prostatic intraepithelial neoplasia (PIN)^29^, our data shows that AR expression is pronounced only in cancer cells but not in PIN lesions in this patient. Again, even though extensive biological conclusion cannot be presented based on a single patient case, this type of tissue cytometry data from solid tumours can only be obtained through *in situ* multiplexed assays.

While the concept of mIHC in FFPE tissues is established, few studies have addressed the compatibility of antibodies for multiplexed IHC assays^2^. As it is important to eliminate false-positive results in assays where the same secondary reagent is applied subsequently (e.g., the commercially available Opal system by PerkinElmer)^22^, we tested whether primary antibodies could be detached from tissues or denatured in situ by incubation in a hot buffer, mimicking the conditions in the HIER procedure during IHC. Surprisingly, we did not observe substantially reduced signal from the anti-CK5/anti-rabbit-AlexaFluor488 or anti-CK8/anti-mouse-AlexaFluor647 antibody complexes when secondary antibodies were applied on the sections before exposing the slides to hot buffer. However, except for E-cadherin and Ki67 antibodies, the signal was completely absent if the slides were exposed to hot buffer before applying secondary antibodies on the sections, even at high antibody concentrations. These findings imply that neither primary nor secondary antibodies are detached from the tissue but the antibody complex is denatured in the tested HIER conditions, and may have implications in mIHC methods relying on antibody stripping. Wahlby *et al*.^2^ showed similar results for nuclear antigens using FITC and CY3 fluorochromes. However, the tissue integrity and antigenicity was compromised as their Sequential Immunofluorescence Staining (SIFS) assay required fluorescence removal after each round of staining by exposing the slides in harsh acidic conditions. In contrast, as our assay design does not require fluorescence removal/bleaching at any step, we can use milder conditions to denature primary antibodies potentially preserving better the epitope antigenicity and tissue integrity. Based on our results, we propose that any antibody showing incomplete denaturation can only be applied in the last round of primary antibodies whenever the same secondary antibody is used more than once during the mIHC assay. We recommend, that the described antibody tests should be performed to verify denaturation of all primary antibodies prior to application in mIHC assays that utilize indirect detection and multiple staining rounds.

Labelling of the primary antibodies for mIHC assays, independent of the imaging modality, complicates assay design. For example, fluorescence dye cycling^5,17^ or mass spectrometry^10–12^ use directly labelled primary antibodies, which require rigorous validation after the labelling reaction. In contrast, mIHC methods that utilize indirect detection employing secondary reagents to detect unlabelled primary antibodies overcome the pitfalls of the labelling issues, but in contrast, are limited in terms of the degree of multiplexing. For all methods utilizing indirect detection, the number of primary antibody host species limited the degree of multiplexing until the invention of tyramide signal amplification (TSA)^18,22^. TSA has stretched the limits of indirect multiplexing by introducing covalent fluorochrome attachment that allows application of primary antibodies from the same host species on multiple subsequent cycles. By using a combination of multispectral imaging and TSA, up to seven targets have been detected in the same tissue section^9,16,22^. However, multispectral imaging is not practical for whole-slide analytics at high resolution across entire tissue specimen. Nevertheless, the most important aspect of indirect detection is the possibility to use commercially available validated primary antibodies without labelling.

Due to strong autofluorescence of formalin-fixed tissues, especially in the blue-green range of the emission spectrum^3^, the fluorescence signal-to-background ratios are not high enough without signal amplification and/or autofluorescence image compensation. We employed TSA for AlexaFluor488 and AlexaFluor555 to overcome tissue autofluorescence in the blue-green spectral region. Additionally, we detected a pair of primary antibodies from different host species using AlexaFluo647 and AlexaFluor750 without TSA, as the tissue autofluorescence is low in the red/infrared region. This approach minimizes heating steps during the assay preserving the tissue integrity and making the staining process faster. With this assay design, we achieved 5-plex fluorescence assay with only two heating steps and 8-plex assay with one additional heating step for a triple colour chromogenic staining (See Supplementary Information for a detailed protocol). This differs from the published methods utilizing TSA^6,8,9,22^, where a heating step is applied prior to every single primary antibody, hence making them slower and potentially compromising the tissue integrity.

Fluorescence channel crosstalk (also known as bleed-through or cross-over) should be addressed when multiple fluorochromes are applied, irrespective of signal intensity. A common solution for reducing crosstalk is unmixing of the individual fluorochrome signals from the mixed image data after image acquisition (also known as de-convolution or bleed-through/cross-over compensation, see Stack *et al*.^8^ for a comprehensive review on the topic). However, an accurate spectral unmixing requires a library of pure fluorescence spectra, which should match the actual multiplexed sample as closely as possible in terms of spectral characteristics and fluorescence intensity. Furthermore, the multispectral imaging produces large datasets, even ten times larger than images acquired using band-pass filters (personal communication with Dr. Bruun at the Institute for Cancer Research, Oslo University Hospital, Norway), which means long image acquisition times and requires intensive computing for analysis, thus limiting the use of these methods for high-resolution whole-slide analytics. In order to increase image acquisition speed and to reduce raw data output as well as to obviate the need for spectral unmixing, we utilized standard optical band-pass filters including NIR detection delivering spectrally resolved raw data already in single image acquisition step for up to five fluorochromes (AlexaFluor488, -555, -647, -750, and Hoechst).

We performed automated whole-slide image analysis using CellProfiler platform^30^. The open-source CellProfiler software is widely used for single cell segmentation and feature extraction, and has been previously applied for tissue image analysis with high efficacy and accuracy^31,32^. Several characteristics of FFPE tissue make the cellular segmentation in image analysis challenging, as has been already discussed earlier^32^. Firstly, the cell morphology is very heterogeneous due to large number of different cell types and partial cutting of the cells in tissue sections. Secondly, the image of a tissue section is only a 2D projection of 3D structure. Thirdly, cells in solid tissue are usually close to each other forming clumps and/or overlapping with each other. However, regardless of the issues with cell segmentation accuracy, automated methods hold many advantages over manual cell detection and classification. Manual tissue image analysis is subjective, often includes large variation between experts, and is impractical when analysing whole tissue sections with hundreds of thousands of cells. For these reasons, we have applied automated image analysis methods. We tested various segmentation algorithms using sub-images of both antibody panels and selected the most accurate segmentation based on expert visual interpretation of the result. The signal-to-noise ratio of nuclei staining was high making the initial segmentation based on nuclei thresholding simple. To design our pipeline as generic as possible, we utilized automatic thresholding (Otsu^33^) instead of manual thresholding. Due to the large tile images with thousands of cells per image, the statistical automatic segmentation avoided failures caused by low cell counts in an image.

As our aim was to design an open-source mIHC platform with easy accessibility and implementation, we designed the cell segmentation pipelines as simple and as generic as possible. Given that image analysis is a compromise between computational complexity, accuracy, and usability, we decided to apply generic object splitting solutions available in CellProfiler based on widely used watershed segmentation from seed points^34^ at both local intensity peaks (nuclei) or at peaks in distance transformed binary image (glands). More complex segmentation methods allow segmentation of overlapping cells^35^, however, most of these methods are computationally demanding. We also experimented with geodesic active contours^36^ for gland segmentation, which did not, however, improve the segmentation result.

In conclusion, we describe an 8-plex mIHC platform for systems pathology enabling quantitative whole-slide analysis at single-cell resolution on FFPE material. The presented platform is not limited in terms of speed, accessibility, or resolution for true whole-slide analysis. Furthermore, as the system is designed for open-source platforms and for a fixed set of secondary reagents, its implementation is straightforward in a regular histopathology laboratory, therefore providing a practical solution for whole-slide mIHC. We believe that the unique combination of 8-plex mIHC assay design, robust whole-slide image acquisition and image analysis, and fully customisable antibody panels makes this system a viable platform for pathology research.

## Methods

### Patient samples

FFPE samples (prostate cancer samples referred as patient 1 and patient 2) were obtained from the Department of Pathology at the Helsinki University Central Hospital (HUCH). Formalin fixation and paraffin embedding were performed in the central laboratory of HUCH according to standard procedures. The samples were anonymous and all patient-related data and unique identifiers were removed, and therefore, the study did not require ethical approval, thus complying with Finnish legislation regulating human tissues obtained for diagnostic purposes (act on the use of human organs and tissue for medical purposes 2.2.2001/101). The FFPE blocks were cut as 3.5 µm sections on Superfrost objective glass slides (Kindler O Gmbh, Germany) using Microm 355 S microtome (Thermo Scientific, Waltham, MA), which were dried overnight at +37 °C and stored at +4 °C. For long-term storage, slides were stored at −20 °C.

### Immunohistochemistry (IHC)

All antibodies used in this study are listed in Table 1. See Supplementary Information for a list of publications for the used antibodies.

**Table 1:** Primary antibodies, fluorochromes, and chromogens used in the study. All antibodies are commercially available. See also Supplementary Figure S5 for primary antibody denaturation testing. AMACR, alpha-methylacyl-CoA racemase; AR, androgen receptor; CK, cytokeratin; EPI, epithelial; HIER, heat-induced epitope retrieval; HRP, horse-radish peroxidase; IC, immune cell; ms, mouse; o/n, overnight; rbt, rabbit; TSA, tyramide signal amplification.

Target	Species	Vendor	Cat number	Dilution^1^	Concentration (µg/ml)	HIER buffer	Label (enzyme)	Incubation	Panel
CK18	ms	SantaCruz	6259	2000	0.1	Tris-EDTA	TSA488	o/n	EPI
CK8	ms	Invitrogen	18–0185z	2000	0.11	Tris-EDTA	TSA555	o/n	EPI
p63	rbt	Abcam	124762	1000	0.87	Tris-EDTA	AlexaFluor647	90 min	EPI
CK5	rbt	Abcam	52635	400 (2000)	0.88	Tris-EDTA	AlexaFluor647	90 min	EPI
E-cadherin	ms	BD biosciences	610182	1000 (10000)	0.25	Tris-EDTA	AlexaFluor750	90 min	EPI
PanCK	ms	Abcam	7753	200 (2000)	5	Tris-EDTA	AlexaFluor750	90 min	EPI
PanCK	ms	Invitrogen	18–0132	200 (2000)	0.18	Tris-EDTA	AlexaFluor750	90 min	EPI
AR	rbt	SantaCruz	13062	2000	0.1	Tris-EDTA	VinaGreen (HRP)	90 min	EPI
AMACR	ms	Abcam	63340	2000	N/A, LOT GR154665-12	Tris-EDTA	Liquid Permanent Red (AP)	90 min	EPI
FoxP3	ms	Abcam	20034	500	2	Tris-EDTA	TSA488	o/n	IC
CD4	ms	Abcam	133616	3000	0.05	Tris-EDTA	TSA555	o/n	IC
CD8	ms	BioSB	5174	100 (1000)	N/A LOT5174JDL05	Tris-EDTA	AlexaFluor750	90 min	IC
CD45	rbt	CellSignalling	13917	100 (500)	N/A, LOT 1	Tris-EDTA	AlexaFluor647	90 min	IC
Ki67	rbt	Abcam	92742	5000	0.14	Tris-EDTA	Liquid Permanent Red (AP)	90 min	IC
E-cadherin	ms	BD biosciences	610182	10000	0.03	Tris-EDTA	VinaGreen (HRP)	90 min	IC
PanCK	ms	Abcam	7753	2000	0.5	Tris-EDTA	VinaGreen (HRP)	90 min	IC
PanCK	ms	Invitrogen	18–0132	2000	0.02	Tris-EDTA	VinaGreen (HRP)	90 min	IC

^1^Brackets indicate the optimized dilution for chromogenic/TSA-amplified detection.

#### Chromogenic IHC

See full protocol in Supplementary Information. Shortly, we removed paraffin from the slides in xylene and subsequent decreasing alcohol series ending in water, and performed heat-induced epitope retrieval (HIER) in hot buffer (+99 °C) for 20 min (PT Module, Thermo Fisher Scientific, Waltham, MA). After HIER, we blocked endogenous peroxide activity in 0.9% H_2_O_2_ solution for 15 min at room temperature (RT). Protein blocking was performed in 10 mM Tris-HCl buffered saline pH 7.4 (TBS) with 10% normal normal goat serum (TBS-NGS) 30 min at RT before primary antibody was applied on the slides. After primary antibodies, we applied anti-mouse or anti-rabbit horseradish peroxidase (HRP) conjugated secondary antibodies (Immunologic, Netherlands) for 30 min at RT to detect primary antibodies. We applied 3,3′-Diaminobenzidine (DAB; Immunologic) according to manufacturer’s instructions for 5 min to visualize antibody complexes and counterstained the slides using 1:10 water dilution of Mayer’s haematoxylin (Dako, Carpinteria, CA) for 1 min. We dehydrated the samples in increasing alcohol series and xylene, applied coverslips, and imaged the slides as described below in the Imaging section.

#### Fluorescence IHC

See Supplementary Information for a full, detailed protocol. We processed the slides as described above, except that after primary antibody, we applied AlexaFluor555 or AlexaFluor647 fluorochrome-conjugated secondary antibodies (Thermo Fisher Scientific) as 1:300 dilutions for 30 min to detect the primary antibody and used 1 µg/ml Hoechst 33342 (Sigma-Aldrich, St. Louis, MO) for nuclear counterstain. After application of coverslips, we imaged the slides as described below in the Imaging section. All antibody dilutions were made in TBS-NGS. We washed the slides three times 5 min at RT after each step in TBS with 0.05% Tween20 (TBST).

### Primary antibody validation for mIHC

#### Fluorochrome heat-stability test

We performed fluorescence IHC as described above by applying anti-cytokeratin 5 (anti-CK5; 1:600 dilution) and anti-cytokeratin 8 (anti-CK8; 1:100 dilution) primary antibodies on prostate tissue sections and detected them using AlexaFluor555-conjugated goat anti-rabbit and AlexaFluor647-conjugated goat anti-mouse secondary antibodies, respectively. After image acquisition (see Imaging section below), slides were incubated in +99 °C Tris-EDTA (pH 9) for 20 minutes and fluorescence images were acquired again. Subsequently, we incubated the slides in +100 °C 25 mM glycine (pH 2) with 2% (w/v) sodium dodecyl sulfate (SDS) for 4 minutes and re-acquired fluorescence images.

#### Antibody testing

We tested all primary antibodies on prostate cancer and lymph node FFPE samples. The antibodies were tested as following: 1) To find optimal concentration, antibodies were tested in different dilutions (typically three to four different dilutions, starting from vendor’s recommendation for IHC) and chromogen staining result was assessed visually. 2) The selected concentration was also used for tyramide signal amplified staining. 3) For non-amplified fluorescence detection, we used five- or ten-fold concentration compared to the concentration used in non-amplified staining. The concentration resulting in higher signal-to-background ratio based on visual assessment was selected as concentration for mIHC.

For heat-induced denaturation, we applied primary antibodies on tissue sections and subsequently heated the slides in +99 °C HIER buffer for 20 minutes. All slides were processed according to the chromogenic IHC protocol as described above, except that slides were heated in hot buffer after primary antibodies were applied on the slides. Heat control slides were stained equally but without the heat exposure.

### Multiplexed immunohistochemistry (mIHC)

The 8-plex mIHC method combined fluorescence and chromogenic detection (Supplementary Fig. S1 and S2). Tissue sections were treated according to the IHC protocol as described above until the application of primary antibodies. See the full and detailed mIHC protocol in Supplementary Information and antibodies used in Table 1. Shortly, after HIER and protein blocking, we applied the first primary antibody and used tyramide signal amplification (TSA) for AlexaFluor488 (PerkinElmer, Waltham, MA) on the slides according to manufacturers instructions. An HRP conjugated host-specific secondary antibody was diluted 1:10 in TBST and incubated for 45 min at RT. After secondary antibody, tyramide reaction was incubated exactly 15 min at RT. The HRP activity was abolished using hydrogen peroxide and sodium azide. After the attenuation of HRP, we performed identical TSA procedure but now with primary antibody from different host and AlexaFluor555 (PerkinElmer). After the second TSA cycle, we performed HIER step for 60 min and applied a pair of primary antibodies raised in different species to detect additional two targets. We detected the primary antibodies with AlexaFluor647 and AlexaFluor750 fluorochrome-conjugated secondary antibodies (Thermo Fisher Scientific) and counterstained nuclei using Hoechst 33342. We applied ProLong Gold mountant (Thermo Fisher Scientific), coverslipped the slides, and acquired whole-slide fluorescence images using five fluorescence channels. After fluorescence imaging, we detached the coverslip and performed another HIER step. After blocking of the slides, we applied a pair of antibodies raised in different species for 90 min at RT. We detected the primary antibodies using alkaline phosphatase (AP) and HRP conjugated secondary antibodies (Immunologic). The secondary antibodies were mixed 1:1 and incubated for 30 min at RT. The antibody complexes were detected using red (Liquid Permanent Red; Dako) and green (VinaGreen; Biocare Medical, Concord, CA) chromogens according to manufacturer´s instructions, respectively. We performed chromogen reactions subsequently for 8 min at RT, first for VinaGreen and then for Liquid Permanent Red. The slides were washed for 1 min in water after each reaction. We counterstained the slides with haematoxylin (1:10 water dilution, 30 s). After mounting (Pertex) and coverslipping, we acquired brightfield images as described below. See Table 1 for antibodies and fluorochromes used in the study.

#### mIHC performance evaluation

Prostate cancer tissue was stained using the mIHC protocol pairwise for FoxP3 (AlexaFluor488) and CD4 (AlexaFluor555) in patient 1, CD4 (AlexaFluor555) and CD45 (AlexaFluor647) in patient 1, and PanEpi (AlexaFluor647), and CK5 + p63 (AlexaFluor750) in patient 2.

### Imaging

All digital, whole-slide fluorescence and brightfield images were acquired at 0.33 µm/pixel or 0.22 µm/pixel resolution, respectively, using Pannoramic P250 Flash II whole-slide scanner (3DHistech, Hungary) equipped with Zeiss Plan-Apochromat 20x objective (NA 0.8) and a modified Spectra X light engine (Lumencor Inc., Beaverton, OR). In the modified light engine the teal LED was replaced with NIR LED, which enabled imaging in the NIR spectral region. The slide scanner stage and optical light-paths were calibrated and aligned by manufacturer (3DHistech) and re-adjusted on-site at scanner installation. The light engine was calibrated at Lumencor. Automatic focusing (default factory settings) was used for both fluorescence and brightfield imaging. For fluorescence imaging, we used DAPI, FITC, CY3, CY5, and CY7 filter sets with compatible LED light sources in the Spectra X engine. See Supplementary Table S1 for optical filters, LED light source specifications, and exposure times used for each fluorochrome/channel. After image acquisition, images were converted to JPEG2000 format (95% quality). Fluorescence images were stored as 8-bit grayscale images and brightfield images as standard RGB images. For mIHC performance evaluation, the stained prostate cancer tissues were imaged as described above using exposure times of 30 ms for FITC channel, 40 ms for CY3 channel, 50 ms or 100 ms for CY5 channel, and 150 ms for CY7 channel.

For the antibody detachment test, snapshot fluorescence images were acquired using AxioImager.Z2 (Zeiss, Germany) microscope equipped with Zeiss Plan-Apochromat 20x objective (NA 0.8), CoolCube1 CCD camera (MetaSystems, Germany), and HXP 120 metal-halide light source. Exposure time was fixed to 300 ms and CY3 and CY5 filter cubes were used for imaging of AlexaFluor555 and AlexaFluor647 fluorochromes, respectively. We selected a representative tissue area manually for the detachment analysis.

### Colour de-convolution and image registration

We separated the different chromogen components in the VinaGreen-LiquidPermanentRed-Haematoxylin-stained samples with a colour de-convolution algorithm^37^ after 1:4 downscaling of the whole-slide images. For registration of the brightfield and fluorescent channels, we applied Speeded-Up Robust Features (SURF)^38^ for keypoint detection and description. In the estimation of the geometric transformation, we used downscaled haematoxylin (1.76 µm/pixels) and DAPI (2.56 µm/pixels) channels, and set the haematoxylin channel as the reference image for descriptor matching. Registered images had an image resolution of 0.64 µm/pixels. Both colour de-convolution and image registration were implemented with a numerical computing environment (MATLAB, MathWorks, Natick, MA, U.S.).

### Image processing, cell segmentation and classification

#### Antibody and fluorochrome heat-stability test

We analysed 8-bit grayscale images using ImageJ64 (1.48 v). We segmented cell objects as following: Image was converted to a binary mask using default setting, after which standard built-in dilation and watershed operations were applied to segment out cell objects. Fluorescence intensity within objects was calculated as mean intensity value per object area (pixels) in objects larger than 100 pixels in size. Data was plotted using Excel for Mac 2011.

#### mIHC performance evaluation

Fluorescence intensities were measured in 8-bit gray-scale images pairwise for neighbouring channels (FITC-CY3, CY3-CY5, and CY5-CY7). We selected areas for measurements with minimal antibody (and fluorochrome) co-localisation in the measured pixels. The average background signal was measured from at least 1,000 visually blank pixels in each image. Signal-to-background ratio (S/B) was defined as background-corrected specific fluorescence intensity (S) divided by the background signal (B). All data was plotted using Excel for Mac 2011.

#### mIHC

We used in-house developed MATLAB scripts for pre- and post-processing of the image data, and CellProfiler 2.1.2 software platform (24) for object segmentation, measurements and classification (Supplementary Fig. S7, code and pipelines available at https://github.com/lopaavol/mihc-suppl-software). As a pre-processing step, the digital whole-slide images (depth 8-bit, resolution 0.64 µm/px) were tiled into 2048 × 2048 pixel regions-of-interests (ROIs) to reduce the memory demand during the image analysis. Since whole epithelial glands were needed for classification of the cells, all ROIs were padded to 10240 × 10240 pixel images. We confirmed that the epithelial glands, which were inside the ROI were also fully included in the padded images.

Next, we segmented epithelial glands and all cells for single cell analysis and classification. First, epithelial glands (Pan-Epi) were segmented using global (Immune cell panel, patient 1) or adaptive (Epithelial cell panel, patient 2) Otsu thresholding (29). Adaptive thresholding was used in epithelial cell analysis to reduce the effect of non-uniform background. Touching glands were separated using watershed segmentation. Second, cells were segmented from the images of nuclei stained channel using adaptive Otsu thresholding. The range for diameter of nucleus was set to 20–80 pixels. Larger clumps were split based on watershed segmentation with intensity peaks as seed points. Third, global Otsu thresholding was used to create a mask from CD45 imaged channel (patient 1) and CK5 + p63 channel (patient 2). After object segmentation, we calculated intensity of all markers and mean radius in the detected cells. The intensity values (scale 0–255) were calculated as mean intensity values per object area (pixels), and were normalized yielding values 0–1 for each marker. A cell was considered positive for a given marker if the expression was higher than one standard deviation (SD) above the mean expression in all cells. For Ki67, we used a threshold three SDs above the mean intensity of all cells to avoid false positive cells due to low number of Ki67^+^ cells in the samples. Cells were automatically classified based on the epithelial gland segmentation and masks for CD45 (patient 1) or CK5 + p63 (patient 2).

In the mIHC of immune cell panel (patient 1), nuclei were first classified either as being inside or outside of an epithelial gland. Based on the CD45 mask image, nuclei were further classified as epithelial leukocytes (inside glands) or stromal leukocytes (outside glands). The rest of the cells inside the glands were classified as epithelial cells and the rest of the cells outside the glands as other cells. CD45^+^ leukocytes were further classified as T regulatory (CD4^+^FoxP3^+^CD8^−^), T helper (CD4^+^FoxP3^−^CD8^−^), or T effector (CD8^+^CD4^–^FoxP3^−^) cells.

In the epithelial cell analysis (patient 2), we classified the glands first either as benign epithelial glands or cancer glands based on the CK5 + p63 expression indicating presence or absence of basal cells in a given gland. Nuclei inside cancer glands were classified as nuclei of cancer cells. Nuclei inside benign epithelial glands were further classified as nuclei of basal cells or benign luminal cells based on the overlap (area overlap minimum 10%) with the CK5 + p63 threshold mask. Nuclei outside glands were classified as nuclei of stromal cells.

After segmentation, measurements and classification of cells, the results and images were stitched together as a whole-slide image and result data. We filtered out cells with centroids in padded region to remove duplicates from the results, and stitched central 2048 × 2048 pixel region from all images together as a whole-slide image for classification visualization. No cells were lost during tiling and stitching as a result of using overlapping padded image regions for analysis.

### Statistical methods

Fisher’s exact test (X^2^, two-tailed, exact method) was used to compare cell counts between two groups. Non-parametric Kolmogorov-Smirnov (KS) test was used to compare marker expressions between two cell populations (non-normally distributed data). Correlation between two continuous variables was calculated using Pearson’s correlation coefficient (r). P-values < 0.05 were considered statistically significant.

### Data availability

The datasets generated during and/or analysed during the current study are available from the corresponding author on reasonable request. Image analysis code and pipelines are available at https://github.com/lopaavol/mihc-suppl-software.

## Electronic supplementary material

Supplementary Information

## References

[1] Camp, R. L., Chung, G. G. & Rimm, D. L. Automated subcellular localization and quantification of protein expression in tissue microarrays. *Nat Med* **8**, 10.1038/nm791 (2002). 10.1038/nm791 12389040

[2] Wahlby C, Erlandsson F, Bengtsson E, Zetterberg A (2002). Sequential immunofluorescence staining and image analysis for detection of large numbers of antigens in individual cell nuclei. Cytometry.

[3] Levenson RM, Mansfield JR (2006). Multispectral imaging in biology and medicine: slices of life. Cytometry. Part A: the journal of the International Society for Analytical Cytology.

[4] Peng C-W (2011). Patterns of cancer invasion revealed by QDs-based quantitative multiplexed imaging of tumor microenvironment. Biomaterials.

[5] Gerdes MJ (2013). Highly multiplexed single-cell analysis of formalinfixed, paraffin-embedded cancer tissue. Proceedings of the National Academy of Sciences of the United States of America.

[6] Brown JR (2014). Multiplexed quantitative analysis of CD3, CD8, and CD20 predicts response to neoadjuvant chemotherapy in breast cancer. Clinical cancer research: an official journal of the American Association for Cancer Research.

[7] Shipitsin M (2014). Automated quantitative multiplex immunofluorescence *in situ* imaging identifies phospho-S6 and phospho-PRAS40 as predictive protein biomarkers for prostate cancer lethality. Proteome Science.

[8] Stack EC, Wang C, Roman KA, Hoyt CC (2014). Multiplexed immunohistochemistry, imaging, and quantitation: A review, with an assessment of Tyramide signal amplification, multispectral imaging and multiplex analysis. Methods.

[9] Carstens JL (2017). Spatial computation of intratumoral T cells correlates with survival of patients with pancreatic cancer. Nat Commun.

[10] Angelo, M. *et al*. Multiplexed ion beam imaging of human breast tumors. *Nat Med* **20**, 436-442, 10.1038/nm.3488http://www.nature.com/nm/journal/v20/n4/abs/nm.3488.html - supplementary-information (2014). 10.1038/nm.3488 PMC4110905 24584119

[11] Giesen C (2014). Highly multiplexed imaging of tumor tissues with subcellular resolution by mass cytometry. Nat Methods.

[12] Levenson RM, Borowsky AD, Angelo M (2015). Immunohistochemistry and mass spectrometry for highly multiplexed cellular molecular imaging. Lab Invest.

[13] Xing Y (2007). Bioconjugated quantum dots for multiplexed and quantitative immunohistochemistry. Nature Protocols.

[14] Mansfield JR (2010). Cellular context in epigenetics: Quantitative multicolor imaging and automated per-cell analysis of miRNAs and their putative targets. Methods.

[15] van der Loos CM (2013). Accurate Quantitation of Ki67-positive Proliferating Hepatocytes in Rabbit Liver by a Multicolor Immunohistochemical (IHC) Approach Analyzed with Automated Tissue and Cell Segmentation Software. Journal of Histochemistry and Cytochemistry.

[16] Feng Z (2015). Multispectral imaging of formalin-fixed tissue predicts ability to generate tumor-infiltrating lymphocytes from melanoma. Journal for ImmunoTherapy of Cancer.

[17] Lin, J.-R., Fallahi-Sichani, M. & Sorger, P. K. Highly multiplexed imaging of single cells using a high-throughput cyclic immunofluorescence method. *Nat Commun* **6**, 10.1038/ncomms9390 (2015). 10.1038/ncomms9390 PMC4587398 26399630

[18] Tóth ZE, Mezey É (2007). Simultaneous Visualization of Multiple Antigens with Tyramide Signal Amplification using Antibodies from the same Species. Journal of Histochemistry & Cytochemistry.

[19] Mansfield, J. R., Hoyt, C. & Levenson, R. M. Visualization of microscopy-based spectral imaging data from multi-label tissue sections. *Curr Protoc Mol Biol* **14** (2008). 10.1002/0471142727.mb1419s84 18972383

[20] Barnes, M. *et al*. Whole tumor section quantitative image analysis maximizes between-pathologists/reproducibility for clinical immunohistochemistry-based biomarkers. *Lab Invest*, 10.1038/labinvest.2017.82 (2017). 10.1038/labinvest.2017.82 28805805

[21] Bobrow MN, Harris TD, Shaughnessy KJ, Litt GJ (1989). Catalyzed reporter deposition, a novel method of signal amplification application to immunoassays. Journal of Immunological Methods.

[22] PerkinElmer. *Opal Multiplex IHC Assay Development Guide*, 2014).

[23] Oleinika K, Nibbs RJ, Graham GJ, Fraser AR (2013). Suppression, subversion and escape: the role of regulatory T cells in cancer progression. Clinical and Experimental Immunology.

[24] Epstein JI (2004). Diagnosis and reporting of limited adenocarcinoma of the prostate on needle biopsy. Mod Pathol.

[25] Prensner JR, Rubin MA, Wei JT, Chinnaiyan AM (2012). Beyond PSA: The next generation of prostate cancer biomarkers. Science translational medicine.

[26] Gaudreau P-O, Stagg J, Soulières D, Saad F (2016). The Present and Future of Biomarkers in Prostate Cancer: Proteomics, Genomics, and Immunology Advancements. Biomarkers in Cancer.

[27] Harper ME, Glynne-Jones E, Goddard L, Mathews P, Nicholson RI (1998). Expression of androgen receptor and growth factors in premalignant lesions of the prostate. The Journal of Pathology.

[28] Walker MM, Ellis SM, Auza MJ, Patel A, Clark P (2007). The intercellular adhesion molecule, cadherin-10, is a marker for human prostate luminal epithelial cells that is not expressed in prostate cancer. Mod Pathol.

[29] Wu CL (2004). Analysis of alpha-methylacyl-CoA racemase (P504S) expression in high-grade prostatic intraepithelial neoplasia. Human pathology.

[30] Kamentsky, L. *et al*. Improved structure, function, and compatibility for CellProfiler: modular high-throughput image analysis software. *Bioinformatics*, 10.1093/bioinformatics/btr095 (2011). 10.1093/bioinformatics/btr095 PMC3072555 21349861

[31] Diem K (2015). Image analysis for accurately counting CD4+ and CD8+ T cells in human tissue. Journal of virological methods.

[32] Xing F, Yang L (2016). Robust Nucleus/Cell Detection and Segmentation in Digital Pathology and Microscopy Images: A Comprehensive Review. IEEE Reviews in Biomedical Engineering.

[33] Otsu N (1979). A Threshold Selection Method from Gray-Level Histograms. IEEE Transactions on Systems, Man, and Cybernetics.

[34] Malpica N (1997). Applying watershed algorithms to the segmentation of clustered nuclei. Cytometry.

[35] Molnar C (2016). Accurate Morphology Preserving Segmentation of Overlapping Cells based on Active Contours. Scientific Reports.

[36] Caselles V, Kimmel R, Sapiro G (1997). Geodesic Active Contours. International Journal of Computer Vision.

[37] Ruifrok AC, Johnston DA (2001). Quantification of histochemical staining by color deconvolution. Analytical and quantitative cytology and histology / the International Academy of Cytology [and] American Society of Cytology.

[38] Bay H, Ess A, Tuytelaars T, Van Gool L (2008). Speeded-Up Robust Features (SURF). Computer Vision and Image Understanding.

