# Supplementary material for: Systems pathology by multiplexed immunohistochemistry and whole-slide digital image analysis
Source: Sci Rep. 2017 Nov 14;7:15580. doi: 10.1038/s41598-017-15798-4 (PMC5686230; doi:10.1038/s41598-017-15798-4)
Supplement: Supplementary file 1 — Supplementary Information [file 41598_2017_15798_MOESM1_ESM.pdf]

Supplementary Information for "Systems pathology by multiplexed immunohistochemistry and whole-slide digital image analysis"

Authors

Sami Blom[1], Lassi Paavolainen[1], Dmitrii Bychkov[1], Riku Turkki[1], Petra Mäki-Teeri[1], Annabrita Hemmes[1], Katja Välimäki[1], Johan Lundin[1], Olli Kallioniemi[1, 2], Teijo Pellinen[1]*

[1]Institute for Molecular Medicine Finland FIMM, University of Helsinki, Helsinki, Finland.

[2]Science for Life Laboratory, Karolinska Institutet, Department of Oncology and Pathology, Solna, Sweden

Supplementary Methods

Immunohistochemistry.

NOTE 1: The full protocol requires a full working day (two days if overnight antibody incubation is included).

NOTE 2: All incubations are performed in a humid chamber.

DAB detection

Instruments

- PT Module, Thermo Fisher Scientific

Reagents

- Xylene, Fluka/FFChemicals
- EtOH, 96%, Altia
- EtOH, 99.8%, Altia
- 10 mM Tris-HCl buffered saline pH 7.4 (TBS), Biotop
- Tris-EDTA buffer (10 mM Tris-HCl, pH 9 + 1 mM EDTA (2-({2-[Bis(carboxymethyl)amino]ethyl}(carboxymethyl)amino)acetic acid), made in house
- H_2O_2, 30%, Fluka
- Normal Goat Serum (NGS), Gibco
- Tween-20, Thermo Fisher Scientific
- Mayer´s Hematoxylin, Dako
- Pertex-mountant, HistoLab
- Secondary antibody, horseradish peroxidase (HRP)-conjugated goat anti-rabbit or anti-mouse antibody, Immunologic

- DAB (3,3'-Diaminobenzidine), Immunologic
- MilliQ-H2O, Millipore

Preparation of working solutions

- 1x TBS + 10 % NGS: Mix 5 ml NGS and 5 ml 10x TBS. Fill with water to 50ml. Filter through 0.22µm. (TBS-NGS)
- 1x TBS + Tween20 buffer: Mix 0.5 ml Tween-20 and 100 ml 10x TBS. Fill with water to 1L, mix well (TBST)

Procedure

Manually

1. Prior IHC, adhere paraffin sections to glass slides overnight at 37°C
2. Deparaffinize sections:
    i. Xylene 3x5 min
    ii. 99.8% EtOH 3x1 min
    iii. 96% EtOH 2x1 min
    iv. 70% EtOH 1x1 min
    v. H_2O for 1x1 min

In PT Module

1. Antigen retrieval: +99°C, 20 min in Tris-EDTA pH9 (buffer valid for two weeks or for 40 slides)

Manually

2. Wash in MilliQ-H_2O, 5 min and in 1x TBS, 5 min
3. Blocking peroxidase activity: Incubate 15 min in 0.9% H_2O_2 solution in TBS
4. Wash 5 min, 1x TBST.

5. Protein blocking: Incubate 15 min in TBS-NGS. Tap the solution off the slide before primary antibody (no washing!)
6. Primary antibody: Diluted in TBS-NGS. Incubate on slides at room temperature (RT)
7. Wash 3 x 5 min, 1x TBST
8. Secondary Antibody (ready-to-use), incubate 30 min at RT
9. Wash 3 x 5 min, 1x TBST
10. DAB stain according to manufacturer's instructions (5 min incubation)
11. Wash 5 min, H_2O
12. Hematoxylin counterstain: 1 min in 10% Mayers Hematoxylin in H_2O
13. Rinse in running tap water, 5 min
14. Dehydration
    i. 70 % EtOH 1x1 min
    ii. 96 % EtOH 2x1 min
    iii. 99.8 % EtOH 3x1 min
    iv. Xylene 3x1 min
15. Mount with Pertex and coverslip

Fluorescence detection

Instruments

- PT Module, Thermo Fisher Scientific

Reagents

- Xylene, Fluka/FFChemicals
- EtOH, 96%, Altia
- EtOH, 99.8%, Altia

- 1x Tris-HCl buffered saline pH 7.4 (TBS), Biotop
- Tris-EDTA buffer (10 mM Tris-HCl, pH 9 + 1 mM EDTA (2-({2-[Bis(carboxymethyl)amino]ethyl}(carboxymethyl)amino)acetic acid), made in house
- H_2O_2, 30%, Fluka
- Normal Goat Serum (NGS), Gibco
- Tween-20, Thermo Fisher Scientific
- Hoechst 33342, Sigma-Aldrich
- Prolong Gold mountant, Thermo Fisher Scientific
- Secondary antibody, goat AlexaFluor555-conjugated anti-rabbit or anti-mouse antibodies, Thermo Fisher Scientific
- MilliQ-H2O, Millipore

Preparation of working solutions

- 1x TBS + 10 % NGS: Mix 5 ml NGS and 5 ml 10x TBS. Fill with water to 50ml. Filter through 0.22μm. (TBS-NGS)
- 1x TBS + Tween20 buffer: Mix 0.5 ml Tween-20 and 100 ml 10x TBS. Fill with water to 1L, mix well (TBST)

Procedure

Manually

1. Prior to IHC, adhere paraffin sections to glass slides overnight at 37°C
2. Deparaffinize sections:
   vi. Xylene 3x5 min
   vii. 99.8% EtOH 3x1 min
   viii. 96% EtOH 2x1 min

ix. 70% EtOH 1x1 min

x. H_2O for 1x1 min

In PT Module

3. Antigen retrieval: +99°C, 20 min in Tris-EDTA pH9 (buffer valid for two weeks or for 40 slides)

Manually

4. Wash in MilliQ-H_2O, 5 min and in 1x TBS, 5 min
5. Blocking peroxidase activity: Incubate 15 min in 0.9% H_2O_2 solution
6. Wash 5 min, 1x TBST.
7. Protein blocking: Incubate 15 min in TBS-NGS. Tap the solution off the slide before primary antibody (no washing!)
8. Primary antibody: Diluted in TBS-NGS. Incubate on slides at room temperature (RT)
9. Wash 3 x 5 min, 1x TBST
10. Secondary antibody 1:300 in TBS-NGS + 1 μg/ml Hoechst, incubate 30 min at RT (protect from light)
11. Wash 3 x 5 min, 1x TBST, rinse 1 min in H_2O
12. Tap excess water off the slide and apply Prolong Gold mountant. Apply coverslip.

Multiplexed immunohistochemistry (mIHC)

NOTE 1: The full protocol requires three working days if overnight antibody incubations are included.

NOTE 2: If needed, the slides may be left overnight (+4°C) in protein blocking buffer at steps 7, 18, or 29.

NOTE 3: All incubations are performed in a humid chamber.

Instruments

- PT Module, Thermo Fisher Scientific

Reagents

- Xylene, Fluka/FFChemicals
- EtOH, 96%, Altia
- EtOH, 99.8%, Altia
- 1x Tris-HCl buffered saline pH 7.4 (TBS), Biotop
- Tris-EDTA buffer (10 mM Tris-HCl, pH 9 + 1 mM EDTA (2-({2-[Bis(carboxymethyl)amino]ethyl}(carboxymethyl)amino)acetic acid), made in house
- H_2O_2, 30%, Fluka
- Normal Goat Serum (NGS), Gibco
- Tween-20, Thermo Fisher Scientific
- Secondary antibody, goat AlexaFluor555 or -647 conjugated anti-rabbit or anti-mouse antibodies, Thermo Fisher Scientific
- Tyramide signal amplification (TSA) kits for AlexaFluore488 and AlexaFluor555, PerkinElmer

- Secondary antibody, horseradish peroxidase (HRP)-conjugated goat anti-rabbit or anti-mouse antibody, Immunologic
- Secondary antibody, alkaline phosphatase (AP)-conjugated goat anti-rabbit or anti-mouse antibody, Immunologic
- Secondary antibody, goat AlexaFluor647- and AlexaFluor750-conjugated anti-rabbit or anti-mouse antibodies, Thermo Fisher Scientific
- VinaGreen (VG), Biocare Medical
- Liquid Permanent Red (LPR), Dako
- Mayer´s Hematoxylin, Dako
- Hoechst 33342, Sigma-Aldrich
- Prolong Gold mountant, Thermo Fisher Scientific
- Pertex-mountant, HistoLab
- MilliQ-H2O, Millipore
- NaN_3, Sigma-Aldrich

Preparation of working solutions

- 1x TBS + 10 % NGS: Mix 5 ml NGS and 5 ml 10x TBS. Fill with water to 50ml. Filter through 0.22µm. (TBS-NGS)
- 1x TBS + Tween20 buffer: Mix 0.5 ml Tween-20 and 100 ml 10x TBS. Fill with water to 1 L, mix well (TBST)

Procedure

Manually

1. Prior to IHC, adhere paraffin sections to glass slides overnight at 37°C
2. Deparaffinize sections:

xi. Xylene 3x5 min

xii. 99.8% EtOH 3x1 min

xiii. 96% EtOH 2x1 min

xiv. 70% EtOH 1x1 min

xv. H_2O for 1x1 min

In PT Module

3. Antigen retrieval: +99°C, 20 min in Tris-EDTA pH9 (buffer valid for two weeks or for 40 slides)

Manually

4. Wash in MilliQ-H_2O, 5 min and in 1x TBS, 5 min
5. Blocking peroxidase activity: Incubate 15 min in 0.9% H_2O_2 solution in TBS
6. Wash 5 min, 1x TBST.
7. Protein blocking: Incubate 15 min in TBS-NGS. Tap the solution off the slide before primary antibody (no washing!)
8. Primary antibody: Diluted in TBS-NGS. Incubate on slides at room temperature (RT).
9. Wash 3 x 5 min, 1x TBST
10. HRP-conjugated secondary antibody 1:10 in TBST, incubate 45 min at RT
11. Wash 3 x 5 min, 1x TBST
12. TSA reaction 1 (and 2):
    i. Prepare 150 µl TSA working solution (AlexaFluor488 or 555) 150 µl per slide according to manufacturer´s instructions
    ii. Incubate exactly 15 min at RT (protect from light)
13. Wash 5 min, 1x TBST

14. Blocking peroxidase activity: Incubate 15 min in 0.9% H_2O_2 solution in TBS with 0.05% NaN_3 (NaN_3 is toxic, handle carefully!)
15. Wash 3 x 5 min, 1x TBST
16. **REPEAT steps 7–13 for TSA reaction 2**, then proceed to step 17
17. Antigen retrieval: +99°C, 20 min in Tris-EDTA pH9 (buffer valid for two weeks or for 40 slides)
18. Protein blocking: Incubate 15 min in TBS-NGS. Tap the solution off the slide before primary antibody (no washing!)
19. Two primary antibodies (rabbit + mouse) diluted in TBS-NGS. Incubate at RT (protect from light)
20. Wash 3 x 5 min, 1x TBST
21. Secondary antibodies (AlexaFluor647 and AlexaFluor750) mixed and diluted 1:300 in TBS-NGS + 1 μg/ml Hoechst, incubate 30 min at RT (protect from light)
22. Wash 3 x 5 min, 1x TBST
23. Tap excess water off the slide, mount with Prolong Gold and apply coverslip
24. IMAGING: Acquire fluorescence images within 4 hours after coverslipping. Continue to next steps as soon as possible after fluorescence imaging!
25. Detach the coverslip by incubating in 1x TBST as long as needed for the coverslip to slide off by gravity (do not force the coverslip off!)
26. Antigen retrieval: +99°C, **60 min** in Tris-EDTA pH 9 (fresh buffer!)
27. Blocking peroxidase activity: Incubate 15 min in 0.9% H_2O_2 solution with 0.05% NaN_3 (NaN_3 is toxic, handle carefully!)

28. Wash 3 x 5 min, 1x TBST
29. Protein blocking: Incubate 15 min in TBS-NGS. Tap the solution off the slide before primary antibody (no washing!)
30. Two primary antibodies (rabbit + mouse) diluted in TBS-NGS. Incubate at RT (protect from light)
31. Secondary antibodies AP- and HRP-conjugated mixed 1:1 in TBS-NGS, incubate 30 min at RT (protect from light)
32. Wash 3 x 5 min, 1x TBST, rinse in H_2O
33. Prepare VG and LPR chromogen working solutions according to manufacturers instructions
    i. Incubate VG 8 min
    ii. Wash **1 min in H_2O**
    iii. Incubate LPR 8 min
    iv. Wash **1 min in H_2O**
34. Hematoxylin counterstain: **30 sec** in 10% Mayers Hematoxylin in H_2O
35. Rinse in tap water **3 min**
36. Let slides dry at RT (do not expose to solvents!)
37. Mount with Pertex mountant and coverslip
38. IMAGING: Acquire brightfield images

Supplementary Figures

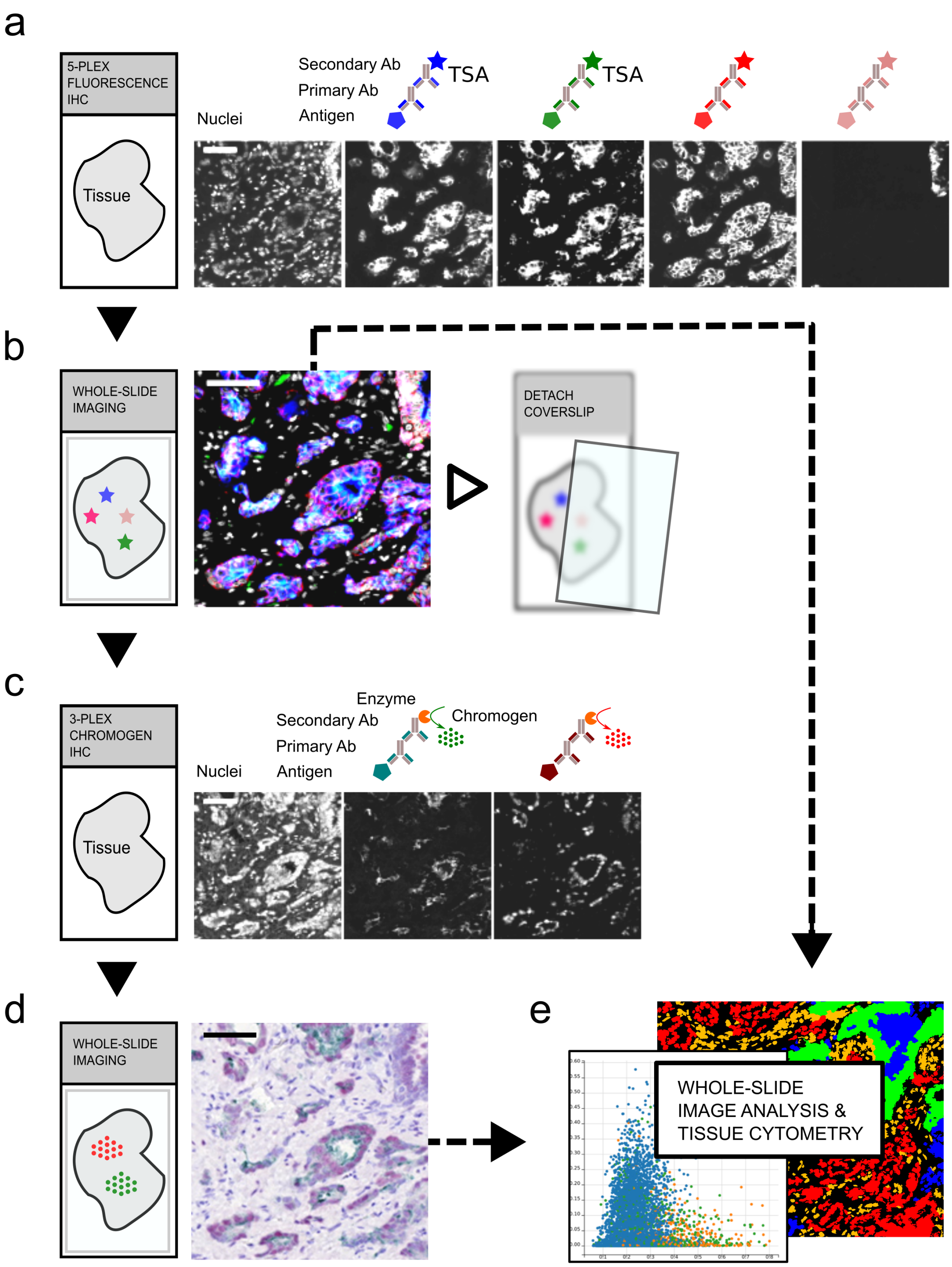

Supplementary Figure S1. Schematic workflow of multiplexed immunohistochemistry protocol. (a) The sectioned tissue is stained for four

targets using two tyramide signal amplification (TSA) reactions and four spectrally distinct fluorochromes with fluorescent nuclear counterstain. (b) The fluorescence image is acquired using high-resolution whole-slide imaging, after which the coverslip is detached. (c) A pair of antibodies is applied and detected using enzyme-linked secondary antibodies with red and green chromogenic substrates and blue counterstain. (d) High-resolution whole-slide transmitted light image is acquired, (e) and data from the fluorescence and the transmitted light images are used whole-slide image analysis. Scale bar 50 μm. Ab, antibody; IHC, immunohistochemistry; TSA, tyramide signal amplification. Relates to Figure 1 and Figure 2.

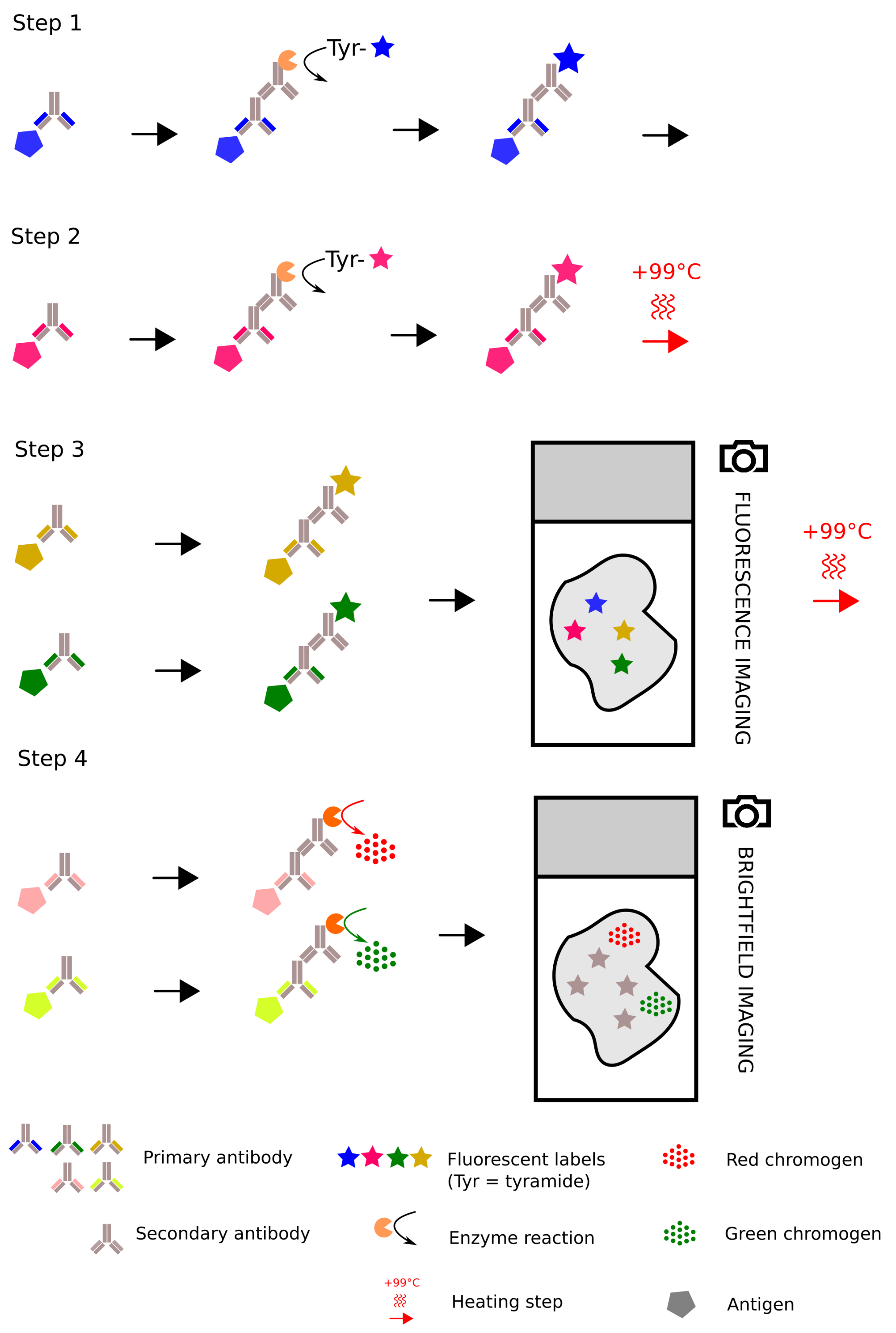

Supplementary Figure S2. Detailed workflow of the multiplexed detection. Steps 1 and 2: Sequential tyramide signal amplification reactions are performed after detecting the primary antibody with HRP-conjugated secondary antibody. The antibody complexes are denatured after the amplification reactions. Step 3: Two

primary antibodies from different hosts are used for a non-amplified fluorescent detection of two additional targets. The fluorescence image is acquired, and antibodies are denatured by heat. Step 4: Two primary antibodies from different hosts are used for an HRP-amplified chromogenic detection of two targets, after which the brightfield image is acquired. Nuclei are counterstained using Hoechst and haematoxylin. Relates to Figure 1 and 2.

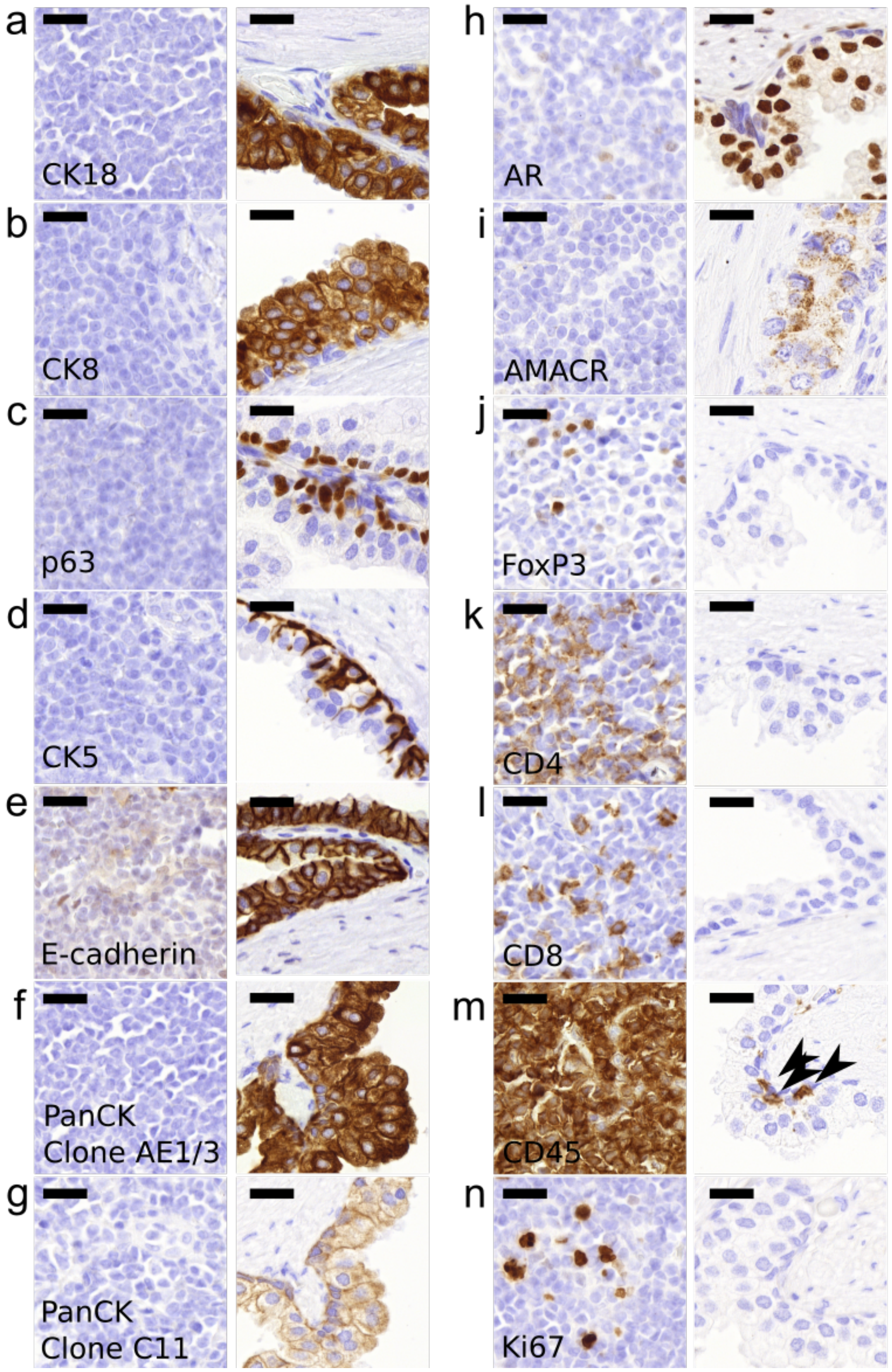

Supplementary Figure S3. Antibody validation. Primary antibodies for (a) CK18, (b) CK8, (c) p63, (d) CK5, (e) E-cadherin, (f) PanCK clone AE1/3 (g) PanCK clone C11, (h) AR, (i) AMACR, (j) FoxP3, (k) CD4, (l) CD8, (m) CD45, (n) Ki67 were tested on lymph node (left panel) and prostate (right panel) FFPE tissues.

Antibody concentrations were the same as optimized for chromogenic or TSA amplified detection (see Table 1). Arrows in (m) indicate CD45+ immune cells in prostate sample.

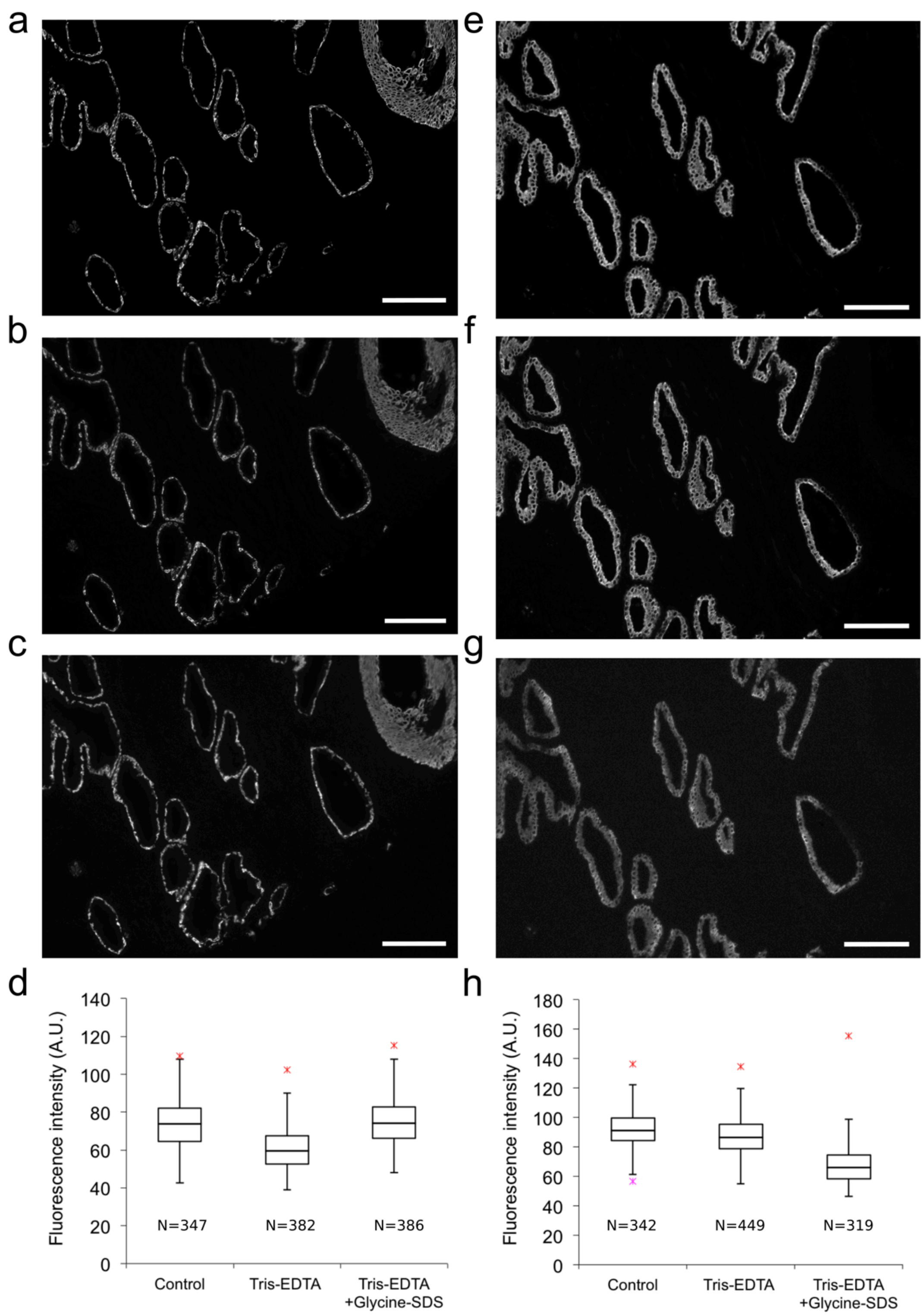

Supplementary Figure S4. Heat-induced antibody detachment test. (a–d) Cytokeratin 5 (CK5) and (e–h) cytokeratin 8 (CK8) were detected using primary

antibodies and AlexaFluor555- or AlexaFluor647-conjugated secondary antibodies, respectively. The fluorescence images were acquired (a, e) before (=control) and (b, f) after heating the slides in hot Tris-EDTA (pH 9) and (c, g) subsequently in hot Glycine-SDS (pH 2). (d, h) Fluorescence intensity in cell object was measured in each condition. Median and quartiles are plotted with maximum and minimum outliers (indicated as cross) A.U., arbitrary unit. Scale bar 100 μm. See Table 1 for information on the primary antibodies.

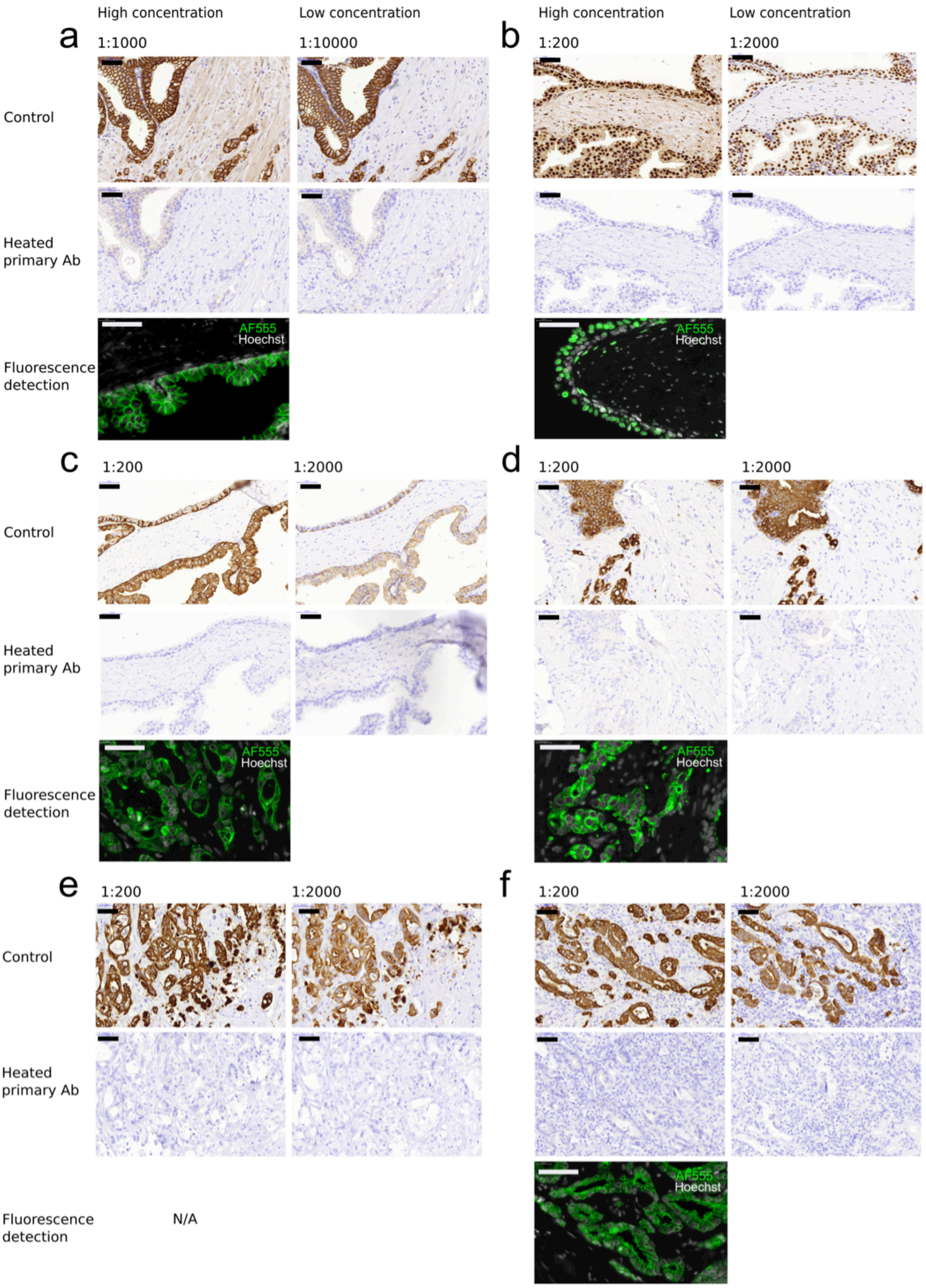

a
High concentration
1:1000
Low concentration
1:10000
b
High concentration
1:200
Low concentration
1:2000
Control
Heated primary Ab
Fluorescence detection
AF555
Hoechst
c
1:200
1:2000
d
1:200
1:2000
e
1:200
1:2000
f
1:200
1:2000
N/A

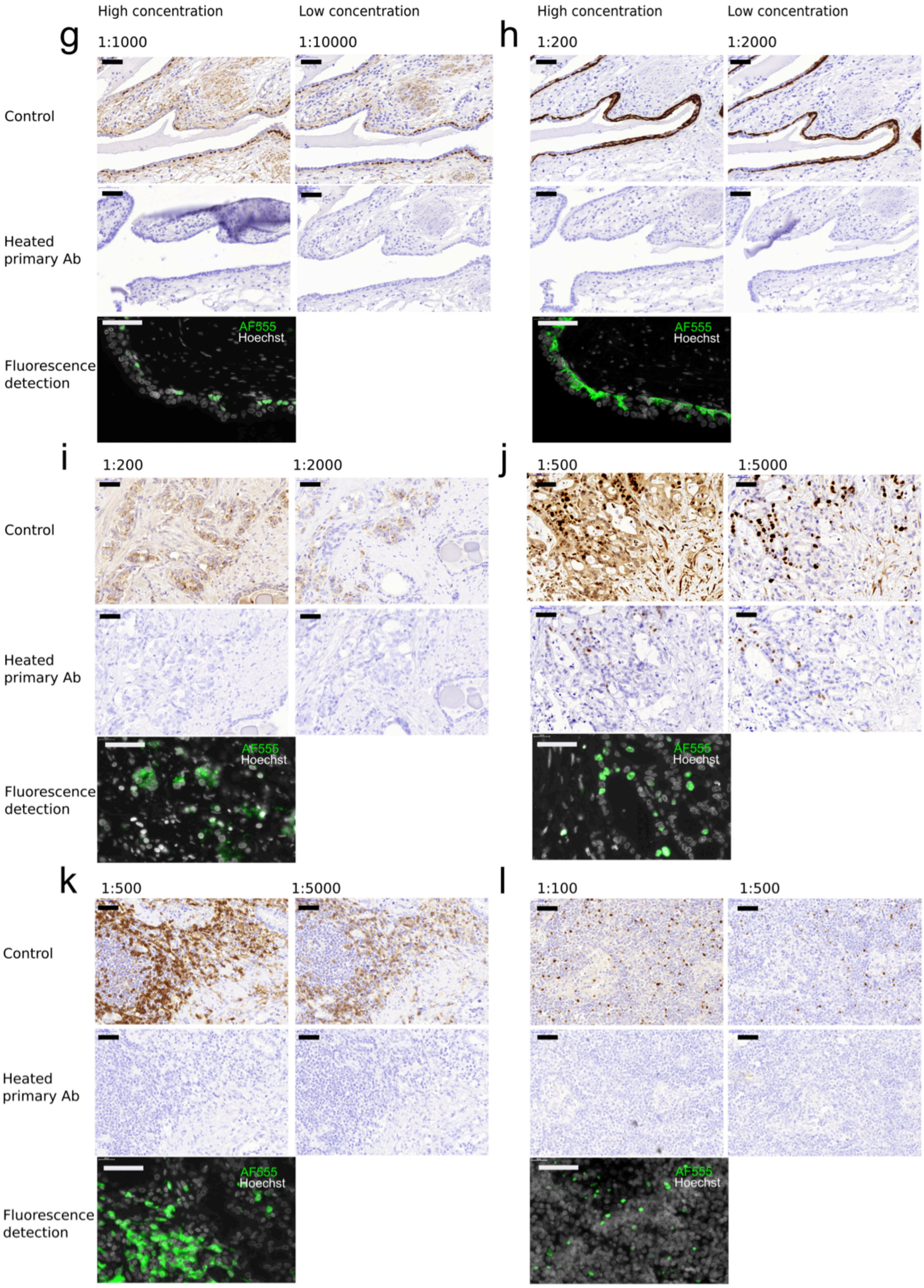

g
High concentration
1:1000
Low concentration
1:10000
h
High concentration
1:200
Low concentration
1:2000
Control
Heated primary Ab
Fluorescence detection
AF555
Hoechst
i
1:200
1:2000
j
1:500
1:5000
k
1:500
1:5000
l
1:100
1:500

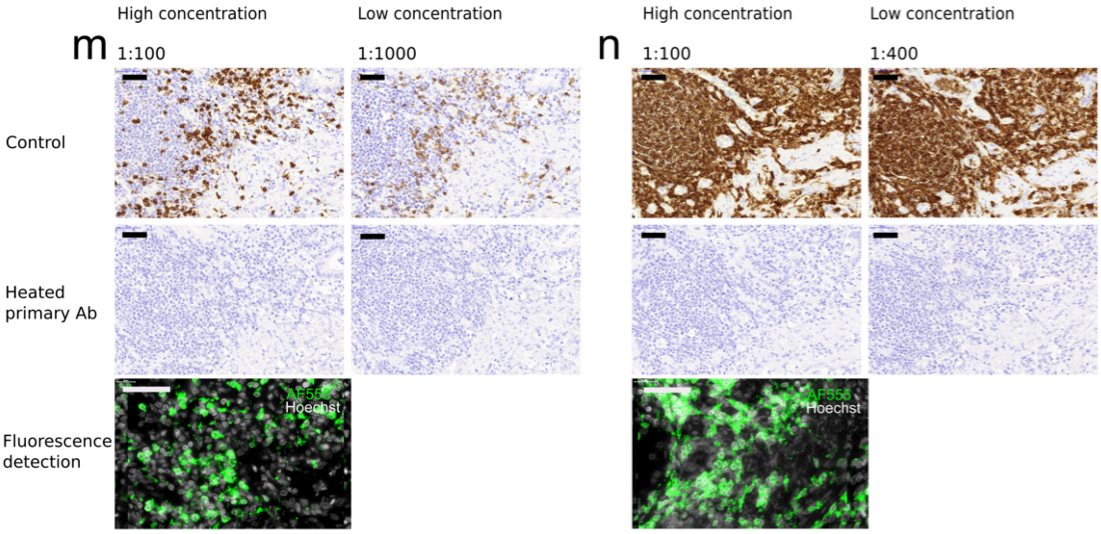

Supplementary Figure S5. Testing of antibodies for heat-induced denaturation. Primary antibodies were tested against (a) E-cadherin, (b) androgen receptor, (c) pan-cytokeratin (Invitrogen), (d) pan-cytokeratin (Abcam), (e) cytokeratin 8, (f) cytokeratin 18, (g) p63, (h) cytokeratin 5, (i) alpha-methylacyl-CoA racemase, (j) Ki67, (k) CD4, (l) FoxP3, (m) CD8, and (n) CD45. The antibodies were tested in two concentrations: Low concentration for amplified detection (TSA or chromogen detection) using HRP-conjugated secondary antibody and high concentration for non-amplified detection using fluorochrome conjugated secondary antibody. Chromogen DAB detection (brown) was used to detect primary antibodies. Any residual DAB signal implies incomplete denaturation of the primary antibody. Fluorescence detection (AlexaFluor555, green) confirmed that staining is adequate for non-amplified detection. (a–j) Prostate cancer and (k–n) lymph node were used as control tissues. N/A, not available. Scale bar 50 µm. See Table 1 for primary antibody details.

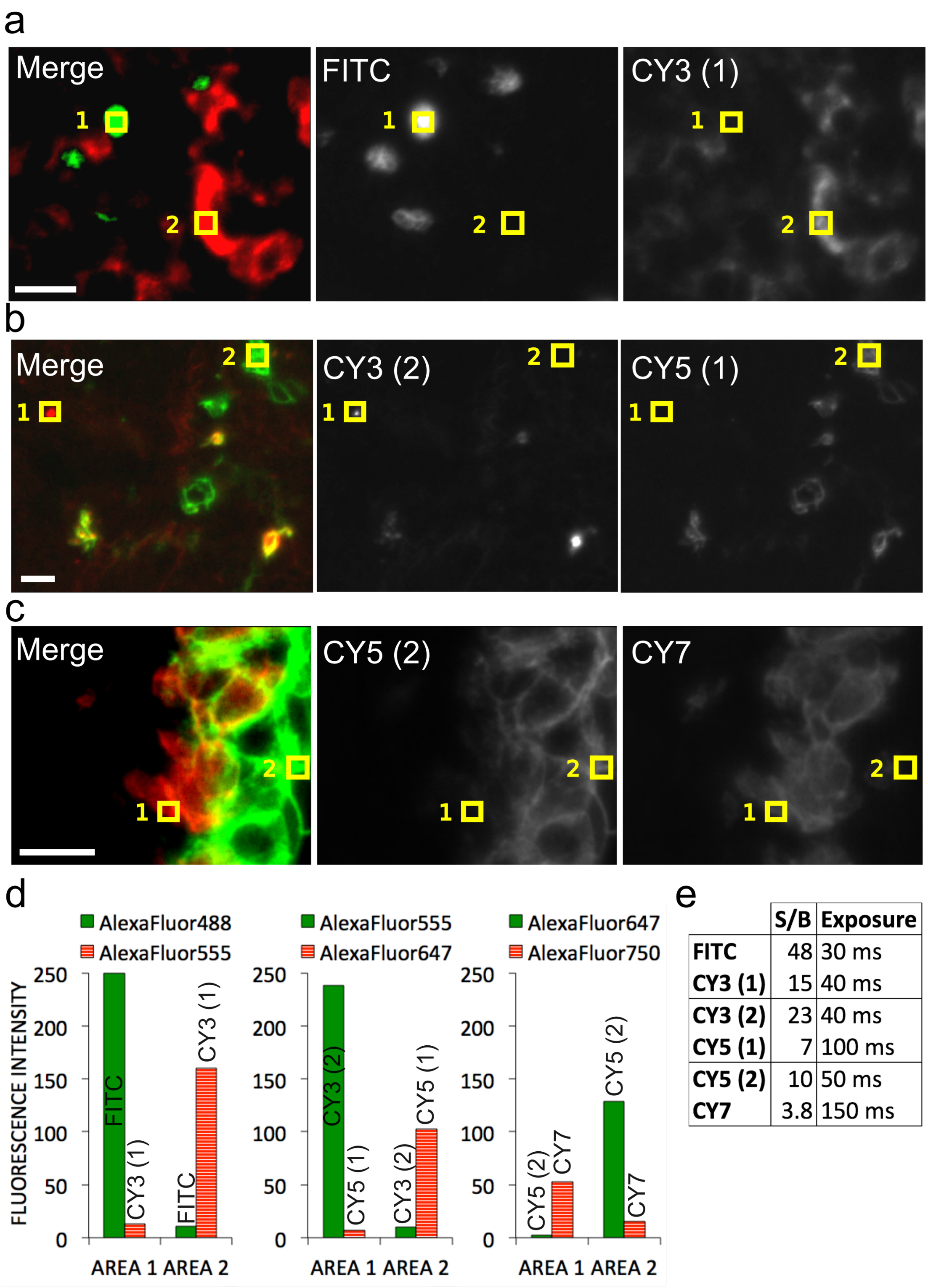

	S/B	Exposure
FITC	48	30 ms
CY3 (1)	15	40 ms
CY3 (2)	23	40 ms
CY5 (1)	7	100 ms
CY5 (2)	10	50 ms
CY7	3.8	150 ms

Supplementary Figure S6. Multiplex fluorescence signal-to-background analysis using mIHC panels with pairwise detection of (a) FoxP3 (AlexaFluor488/FITC) and CD4 (AlexaFluor555/CY3(1)), (b) CD4 (AlexaFluor555/CY3(2)) and CD45 (AlexaFluor647/CY5(1)) in patient 1, and (c) PanEpi (AlexaFluor647/CY5(2)) and CK5+p63 (AlexaFluor750/CY7) in patient 2. (d) Background-corrected

fluorescence intensities were measured pairwise in neighbouring channels for FITC-CY3, CY3-CY5, and CY5-CY7 in two areas (area 1 and area 2), where the antibodies (and fluorochromes) are not co-localizing in the measured pixels (visual assessment). (e) Corresponding signal-to-background ratios (area1/area2 or area2/area1) with indicated exposure times. Scale bar 10 µm. S/B, signal-to-background; ms, millisecond.

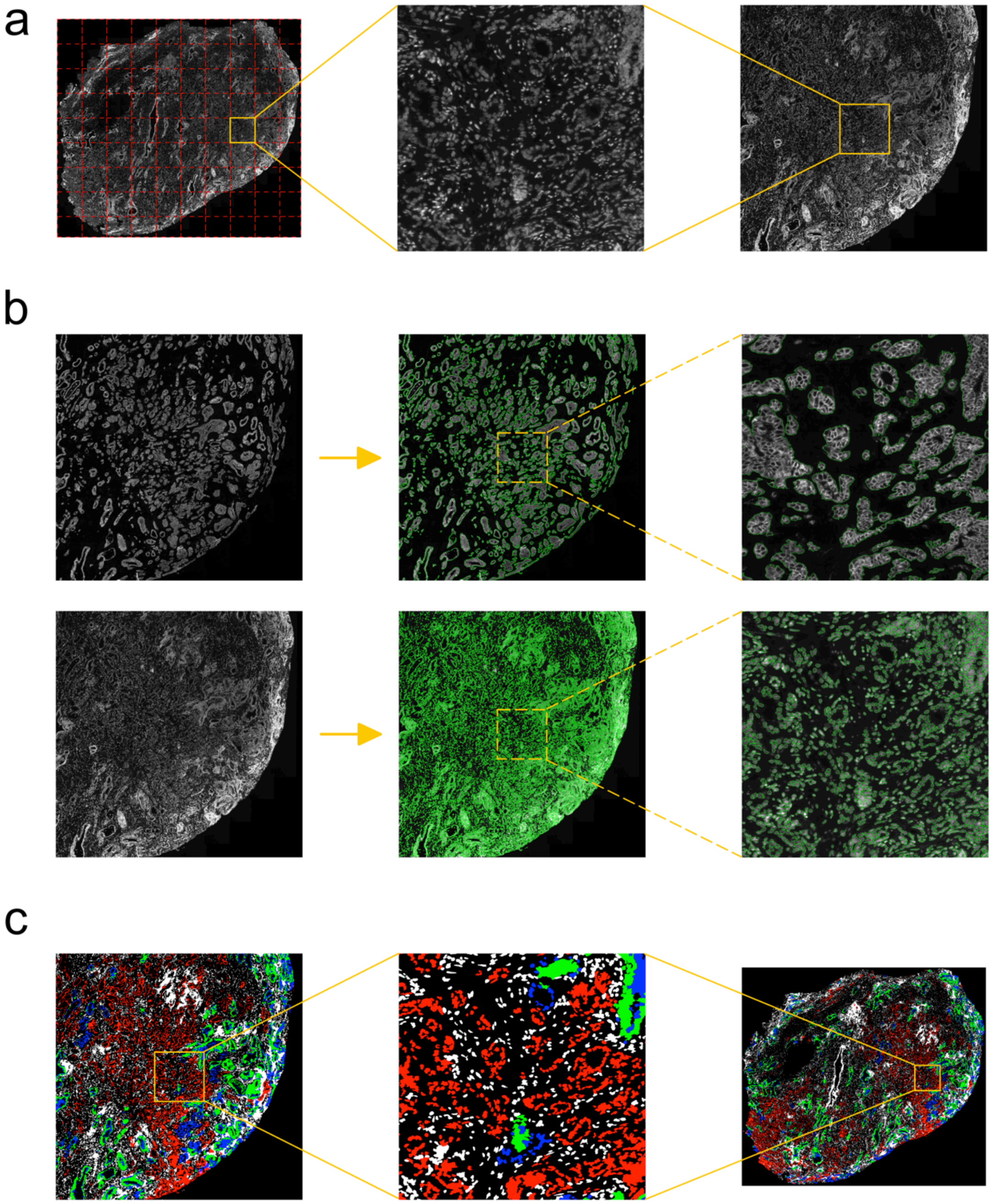

Supplementary Figure S7. Image analysis workflow. (a) Whole slide images of all imaged channels were split into 2048x2048 pixel subregions, which were padded to 10240x10240 pixel image to enable segmentation of whole prostatic glands (used later for cell classification). Every marker in the padded image outside the whole slide image was set to zero, and the padded images were set as input for the image analysis pipeline. (b) Cells and glands were segmented from

padded input images. The intensity of each marker is measured inside every segmented cell (segmentation result of cells and glands is shown for visualization purposes only, the subregion is not extracted at this point in the workflow). (c) Every cell in the padded image is classified based on the marker profile and the gland segmentation. The central 2048x2048 pixel subregion of each padded image is extracted with its cell measurements (a cell belongs to the subregion where its centroid is located) and used to stitch the classified whole slide image with its cell measurements. Relates to Figure 1 and 2.

Supplementary tables

Supplementary Table S1. Fluorochromes and fluorescence imaging specifications. LED light source was used for the excitation of all fluorochromes. ms, millisecond; mW, milliwatt.

Fluorochrome	Filter set	Excitation filter	Dichroic mirror	Emission filter	Excitation light source (power)	Exposure time, immune panel	Exposure time, epithelial panel
Hoechst 33342	DAPI cube (Zeiss Filter Set 02)	G365	FT395	LP420	390/18 (264 mW)	50 ms	50 ms
Tyramide-AlexaFluor488	FITC cube (Zeiss Filter Set 38 HE)	BP470/40 (HE)	FT495 (HE)	BP525/50 (HE)	475/28 (191 mW)	30 ms	20 ms
Tyramide-AlexaFluor555	Cy3 cube (Chroma Technology Corp 49004 ET CY3/R)	ET545/25x	T565lpxr	ET605/70m	542/27 (403 mW)	40 ms	100 ms
AlexaFluor647	Cy5 cube (Chroma Technology Corp 49006 ET CY5)	ET620/60x	T660lpxr	ET700/75m	633/22 (143 mW)	100 ms	50 ms
AlexaFluor750	Cy7 cube (Chroma Technology Corp 49007 ET CY7)	ET710/75x	T760lpxr	ET810/90m	740/40 (140 mW)	300 ms	150 ms

Supplementary Table S2. Normalized expression of markers in different cell classes using immune cell panel and epithelial antibody panel (range 0–1). AMACR, alpha-methylacyl-CoA racemase; AR, androgen receptor; ECad, epithelial cadherin; Pan-Epi, pan-epithelium; SD, standard deviation. Relates to Figures 3 and 4.

**Immune cell panel**	**Epithelial cells**		**Epithelial leukocytes**		**Stromal leukocytes**		**Other cells**		**All cells**		**Positivity threshold**
**Marker**	**Mean**	**SD**	**Mean**	**SD**	**Mean**	**SD**	**Mean**	**SD**	**Mean**	**SD**	
Pan-Epi	0.10	0.07	0.13	0.09	0.00	0.00	0.01	0.01	0.06	0.07	Global Otsu
CD45	0.02	0.00	0.07	0.05	0.13	0.06	0.03	0.01	0.06	0.06	Global Otsu
CD8	0.07	0.00	0.08	0.01	0.08	0.01	0.07	0.01	0.08	0.01	0.09
CD4	0.02	0.01	0.05	0.06	0.08	0.08	0.02	0.02	0.04	0.05	0.09
FoxP3	0.01	0.00	0.02	0.04	0.03	0.07	0.01	0.01	0.02	0.04	0.06
Ki67	0.01	0.03	0.01	0.02	0.01	0.02	0.01	0.02	0.01	0.03	0.10
**Epithelial cell panel**	**Cancer cells**		**Benign luminal cells**		**Basal cells**		**Stromal cells**		**All cells**		**Positivity threshold**
**Marker**	**Mean**	**SD**	**Mean**	**SD**	**Mean**	**SD**	**Mean**	**SD**	**Mean**	**SD**	
Pan-Epi	0.139	0.062	0.127	0.072	0.176	0.061	0.021	0.027	0.079	0.080	Adaptive Otsu
CK5 + p63	0.040	0.003	0.048	0.008	0.104	0.037	0.043	0.010	0.051	0.027	Global Otsu
CK8	0.081	0.022	0.071	0.023	0.061	0.014	0.052	0.013	0.061	0.021	0.082
CK18	0.200	0.153	0.119	0.136	0.075	0.089	0.017	0.017	0.073	0.115	0.188
AMACR	0.038	0.052	0.011	0.023	0.006	0.007	0.005	0.006	0.013	0.028	0.041
AR	0.143	0.106	0.102	0.089	0.084	0.063	0.056	0.048	0.083	0.078	0.161

Supplementary Table S3. Summary of publications for multiplexed IHC. None of the published methods fulfil all the specifications listed. Relates to Figure 5.

Reference	Publication year	Reference nr	>5-plex assay	Rapid implementation of new targets	High-resolution whole-slide image acquisition	High-resolution whole-slide image analysis	Open-source image analysis software
Camp, R. L., Chung, G. G. & Rimm, D. L.	2002	1	No	Yes	No	No	Yes
Wahlby, C., Erlandsson, F., Bengtsson, E. & Zetterberg, A.	2002	2	Yes	Yes	No	No	Yes
Xing, Y. *et al.*	2007	13	No	Yes	No	No	Yes
Mansfield, J. R.	2010	14	No	Yes	No	No	No
Peng, C.-W. et al.	2011	4	No	Yes	No	No	No
Gerdes, M. J. *et al.*	2013	5	Yes	No	Yes	No	Yes
van der Loos, C. M. *et al.*	2013	15	No	Yes	No	No	No
Angelo, M. *et al.*	2014	10	Yes	No	No	No	Yes
Brown, J. R. *et al.*	2014	6	No	Yes	No	No	No
Giesen, C. *et al.*	2014	11	Yes	No	No	No	Yes
Shipitsin, M. *et al.*	2014	7	No	Yes	No	No	No
Lin, J.-R., Fallahi-Sichani, M. & Sorger, P. K.	2015	17	Yes	No	No	No	Yes
Feng, Z. *et al.*	2015	16	Yes	Yes	No	No	No
Carstens, J. L. et al.	2017	9	Yes	Yes	No	No	No

Supplementary data on publications for antibodies used in this study. IHC, immunohistochemistry; ICC, immunocytochemistry; IF, immunofluorescence; LMA, lysate microarray.

CK18 (SantaCruz 6259, clone DC10)

Original publication

Lauerová L, Kovarik J, Bártek J, Rejthar A, Vojtěsek B. Novel monoclonal antibodies defining epitope of human cytokeratin 18 molecule. Hybridoma. 1988 Oct;7(5):495-504. PubMed PMID: 2461901.

IHC

Jiang LW, Chen H, Lu H. Using human epithelial amnion cells in human de-epidermized dermis for skin regeneration. J Dermatol Sci. 2016 Jan;81(1):26-34. doi: 10.1016/j.jdermsci.2015.10.018. Epub 2015 Oct 31. PubMed PMID: 26596214.

ICC/IHC

Freyer N, Knöspel F, Strahl N, Amini L, Schrade P, Bachmann S, Damm G, Seehofer D, Jacobs F, Monshouwer M, Zeilinger K. Hepatic Differentiation of Human Induced Pluripotent Stem Cells in a Perfused Three-Dimensional Multicompartment Bioreactor. Biores Open Access. 2016 Aug 1;5(1):235-48. doi: 10.1089/biores.2016.0027. eCollection 2016. PubMed PMID: 27610270; PubMed Central PMCID: PMC5003005.

CK8 (Invitrogen 18-0185, clone 5D3)

IHC

Duret C, Gerbal-Chaloin S, Ramos J, Fabre JM, Jacquet E, Navarro F, Blanc P, Sa-Cunha A, Maurel P, Daujat-Chavanieu M. Isolation, characterization, and differentiation to hepatocyte-like cells of nonparenchymal epithelial cells from adult human liver. Stem Cells. 2007 Jul;25(7):1779-90. Epub 2007 Apr 5. PubMed PMID: 17412893.

p63 (Abcam 124762, clone EPR5701)

IHC

Smirnova NF, Schamberger AC, Nayakanti S, Hatz R, Behr J, Eickelberg O. Detection and quantification of epithelial progenitor cell populations in human healthy and IPF lungs. Respir Res. 2016 Jul 16;17(1):83. doi: 10.1186/s12931-016-0404-x. PubMed PMID: 27423691; PubMed Central PMCID: PMC4947297.

CK5 (Abcam 52635, clone EP1601Y

IHC

Volkmer JP, Sahoo D, Chin RK, Ho PL, Tang C, Kurtova AV, Willingham SB, Pazhanisamy SK, Contreras-Trujillo H, Storm TA, Lotan Y, Beck AH, Chung BI,

Alizadeh AA, Godoy G, Lerner SP, van de Rijn M, Shortliffe LD, Weissman IL, Chan KS. Three differentiation states risk-stratify bladder cancer into distinct subtypes. Proc Natl Acad Sci U S A. 2012 Feb 7;109(6):2078-83. doi: 10.1073/pnas.1120605109. Epub 2012 Jan 19. Erratum in: Proc Natl Acad Sci U S A. 2012 Feb 28;109(9):3600. PubMed PMID: 22308455; PubMed Central PMCID: PMC3277552.

E-cadherin (BD Biosciences 610182, clone 36)

Western blot / IF

Weng Z, Xin M, Pablo L, Grueneberg D, Hagel M, Bain G, Müller T, Papkoff J. Protection against anoikis and down-regulation of cadherin expression by a regulatable beta-catenin protein. J Biol Chem. 2002 May 24;277(21):18677-86. Epub 2002 Mar 19. PubMed PMID: 11904289.

IHC

Krebs AM, Mitschke J, Losada ML, Schmalhofer O, Boerries M, Busch H, Boettcher M, Mougiakakos D, Reichardt W, Bronsert P, Brunton VG, Pilarsky C, Winkler TH, Brabletz S, Stemmler MP, Brabletz T. The EMT-activator Zeb1 is a key factor for cell plasticity and promotes metastasis in pancreatic cancer. Nat Cell Biol. 2017 Apr 17. doi: 10.1038/ncb3513. [Epub ahead of print] PubMed PMID: 28414315.

PanCK (Abcam 7753, clone C-11)

Original publication

Bártek J, Vojtěsek B, Stasková Z, Bártková J, Kerekés Z, Rejthar A, Kovarík J. A series of 14 new monoclonal antibodies to keratins: characterization and value in diagnostic histopathology. J Pathol. 1991 Jul;164(3):215-24. PubMed PMID: 1716305.

IHC

Yadav A, Kumar B, Datta J, Teknos TN, Kumar P. IL-6 promotes head and neck tumor metastasis by inducing epithelial-mesenchymal transition via the JAK-STAT3-SNAIL signaling pathway. *Molecular cancer research : MCR*. 2011;9(12):1658-1667. doi:10.1158/1541-7786.MCR-11-0271.

PanCK (Invtrogen 18-0132, clone AE1/3)

IHC

Yang L, Jung Y, Omenetti A, et al. Fate-Mapping Evidence that Hepatic Stellate Cells are Epithelial Progenitors in Adult Mouse Livers. *Stem cells (Dayton, Ohio)*. 2008;26(8):2104-2113. doi:10.1634/stemcells.2008-0115.

Western blot / IHC

Gao D, Vela I, Sboner A, Iaquinta PJ, Karthaus WR, Gopalan A, Dowling C, Wanjala JN, Undvall EA, Arora VK, Wongvipat J, Kossai M, Ramazanoglu S, Barboza LP, Di W, Cao Z, Zhang QF, Sirota I, Ran L, MacDonald TY, Beltran H, Mosquera JM,

Touijer KA, Scardino PT, Laudone VP, Curtis KR, Rathkopf DE, Morris MJ, Danila DC, Slovin SF, Solomon SB, Eastham JA, Chi P, Carver B, Rubin MA, Scher HI, Clevers H, Sawyers CL, Chen Y. Organoid cultures derived from patients with advanced prostate cancer. Cell. 2014 Sep 25;159(1):176-87. doi: 10.1016/j.cell.2014.08.016. Epub 2014 Sep 4. PubMed PMID: 25201530; PubMed Central PMCID: PMC4237931.

AR (H-280) (SantaCruz 13062)

LMA

Östling P, Leivonen SK, Aakula A, Kohonen P, Mäkelä R, Hagman Z, Edsjö A, Kangaspeska S, Edgren H, Nicorici D, Bjartell A, Ceder Y, Perälä M, Kallioniemi O. Systematic analysis of microRNAs targeting the androgen receptor in prostate cancer cells. Cancer Res. 2011 Mar 1;71(5):1956-67. doi: 10.1158/0008-5472.CAN-10-2421. Epub 2011 Feb 22. PubMed PMID: 21343391.

IHC / Western blot

Guo Z, Yang X, Sun F, Jiang R, Linn DE, Chen H, Chen H, Kong X, Melamed J, Tepper CG, Kung HJ, Brodie AM, Edwards J, Qiu Y. A novel androgen receptor splice variant is up-regulated during prostate cancer progression and promotes androgen depletion-resistant growth. Cancer Res. 2009 Mar 15;69(6):2305-13. doi: 10.1158/0008-5472.CAN-08-3795. Epub 2009 Feb 24. PubMed PMID: 19244107; PubMed Central PMCID: PMC2672822.

AMACR (Abcam 63340, clone 2A10F3)

No references.

FoxP3 (Abcam 20034, clone 236A/E7)

IHC

Nagata Y, Kontani K, Enami T, Kataoka K, Ishii R, Totoki Y, Kataoka TR, Hirata M, Aoki K, Nakano K, Kitanaka A, Sakata-Yanagimoto M, Egami S, Shiraishi Y, Chiba K, Tanaka H, Shiozawa Y, Yoshizato T, Suzuki H, Kon A, Yoshida K, Sato Y, Sato-Otsubo A, Sanada M, Munakata W, Nakamura H, Hama N, Miyano S, Nureki O, Shibata T, Haga H, Shimoda K, Katada T, Chiba S, Watanabe T, Ogawa S. Variegated RHOA mutations in adult T-cell leukemia/lymphoma. Blood. 2016 Feb 4;127(5):596-604. doi: 10.1182/blood-2015-06-644948. Epub 2015 Nov 16. PubMed PMID: 26574607; PubMed Central PMCID: PMC5291304.

IHC

Ward ST, Li KK, Hepburn E, Weston CJ, Curbishley SM, Reynolds GM, Hejmadi RK, Bicknell R, Eksteen B, Ismail T, Rot A, Adams DH. The effects of CCR5 inhibition on regulatory T-cell recruitment to colorectal cancer. Br J Cancer. 2015 Jan 20;112(2):319-28. doi: 10.1038/bjc.2014.572. Epub 2014 Nov 18. PubMed PMID: 25405854; PubMed Central PMCID: PMC4301825.

IHC

Lai C, August S, Albibas A, Behar R, Cho SY, Polak ME, Theaker J, MacLeod AS, French RR, Glennie MJ, Al-Shamkhani A, Healy E. OX40+ Regulatory T Cells in Cutaneous Squamous Cell Carcinoma Suppress Effector T-Cell Responses and Associate with Metastatic Potential. Clin Cancer Res. 2016 Aug 15;22(16):4236-48. doi: 10.1158/1078-0432.CCR-15-2614. Epub 2016 Mar 31. PubMed PMID: 27034329; PubMed Central PMCID: PMC4987192.

CD4 (Abcam 133616, clone EPR6855)

IHC

d'Ettorre G, Baroncelli S, Micci L, Ceccarelli G, Andreotti M, Sharma P, Fanello G, Fiocca F, Cavallari EN, Giustini N, Mallano A, Galluzzo CM, Vella S, Mastroianni CM, Silvestri G, Paiardini M, Vullo V. Reconstitution of intestinal CD4 and Th17 T cells in antiretroviral therapy suppressed HIV-infected subjects: implication for residual immune activation from the results of a clinical trial. PLoS One. 2014 Oct 23;9(10):e109791. doi: 10.1371/journal.pone.0109791. eCollection 2014. PubMed PMID: 25340778; PubMed Central PMCID: PMC4207675.

IHC

Schuhmann MK, Gunreben I, Kleinschnitz C, Kraft P. Immunohistochemical Analysis of Cerebral Thrombi Retrieved by Mechanical Thrombectomy from Patients with Acute Ischemic Stroke. Int J Mol Sci. 2016 Feb 26;17(3):298. doi:

10.3390/ijms17030298. PubMed PMID: 26927082; PubMed Central PMCID: PMC4813162.

CD8 (BioSB 5147, clone C8/144B)

No references specific for the BioSB antibody. References for clone C8/144B.

IHC

Chen PL, Roh W, Reuben A, Cooper ZA, Spencer CN, Prieto PA, Miller JP, Bassett RL, Gopalakrishnan V, Wani K, De Macedo MP, Austin-Breneman JL, Jiang H, Chang Q, Reddy SM, Chen WS, Tetzlaff MT, Broaddus RJ, Davies MA, Gershenwald JE, Haydu L, Lazar AJ, Patel SP, Hwu P, Hwu WJ, Diab A, Glitza IC, Woodman SE, Vence LM, Wistuba II, Amaria RN, Kwong LN, Prieto V, Davis RE, Ma W, Overwijk WW, Sharpe AH, Hu J, Futreal PA, Blando J, Sharma P, Allison JP, Chin L, Wargo JA. Analysis of Immune Signatures in Longitudinal Tumor Samples Yields Insight into Biomarkers of Response and Mechanisms of Resistance to Immune Checkpoint Blockade. Cancer Discov. 2016 Aug;6(8):827-37. doi: 10.1158/2159-8290.CD-15-1545. Epub 2016 Jun 14. PubMed PMID: 27301722; PubMed Central PMCID: PMC5082984.

IHC

Ruffell B, Au A, Rugo HS, Esserman LJ, Hwang ES, Coussens LM. Leukocyte composition of human breast cancer. Proc Natl Acad Sci U S A. 2012 Feb

21;109(8):2796-801. doi: 10.1073/pnas.1104303108. Epub 2011 Aug 8. PubMed PMID: 21825174; PubMed Central PMCID: PMC3287000.

CD45 (Cell Signaling 13917, clone D9M8I)

No publications.

Ki67 (Abcam 92742, clone EPR3610)

IHC

Stock K, Estrada MF, Vidic S, Gjerde K, Rudisch A, Santo VE, Barbier M, Blom S, Arundkar SC, Selvam I, Osswald A, Stein Y, Gruenewald S, Brito C, van Weerden W, Rotter V, Boghaert E, Oren M, Sommergruber W, Chong Y, de Hoogt R, Graeser R. Capturing tumor complexity in vitro: Comparative analysis of 2D and 3D tumor models for drug discovery. Sci Rep. 2016 Jul 1;6:28951. doi: 10.1038/srep28951. PubMed PMID: 27364600; PubMed Central PMCID: PMC4929472.

IHC

Qin Q, Wei F, Zhang J, Wang X, Li B. miR-134 inhibits non-small cell lung cancer growth by targeting the epidermal growth factor receptor. J Cell Mol Med. 2016 Oct;20(10):1974-83. doi: 10.1111/jcmm.12889. Epub 2016 May 31. PubMed PMID: 27241841; PubMed Central PMCID: PMC4891324.
